# A 3D lymph node model for chronic lymphocytic leukaemia recapitulates microenvironmental features and drug response *in vitro*

**DOI:** 10.1242/dmm.052731

**Published:** 2026-03-31

**Authors:** Daniela Belloni, Dafne Barozzi, Giulia Milani, Federica Barbaglio, Aswin Raj Puthukkunnath, Pamela Ranghetti, Eleonora Perotta, Teresa Musco, Marta Sampietro, Maurilio Ponzoni, Lydia Scarfò, Paolo Ghia, Cristina Scielzo

**Affiliations:** ^1^ Unit of Malignant B Cells Biology and 3D Modelling, Division of Experimental Oncology, IRCCS Ospedale San Raffaele, Comprehensive Cancer Center, 20132 Milan, Italy; ^2^ Unit B Cell Neoplasia, Division of Experimental Oncology, IRCCS Ospedale San Raffaele, Comprehensive Cancer Center, 20132 Milan, Italy; ^3^ Medical School, Università Vita-Salute San Raffaele, 20132 Milan, Italy; ^4^ Strategic Research Program on CLL, IRCCS Ospedale San Raffaele, 20132 Milan, Italy; ^5^ Pathology Unit, IRCCS Ospedale San Raffaele, 20132 Milan, Italy

**Keywords:** 3D cell cultures, Chronic lymphocytic leukaemia, Lymph node, Targeted therapies

## Abstract

Chronic lymphocytic leukaemia (CLL) cells circulate between the blood, bone marrow (BM) and lymphoid organs, where interactions with the lymph node (LN) microenvironment enhance their survival, proliferation and drug resistance. Most *in vitro* models fail to reproduce the spatial and cellular complexity of the LN niche, limiting studies of tissue-specific drug responses. To address this, we developed a 3D LN model using a gelatine scaffold and a clinorotator bioreactor previously validated for a BM system. The scaffold was seeded with human lymphatic fibroblasts and endothelial cells, which deposited extracellular matrix and supported patient-derived CLL cell viability and proliferation. Consistent with *in vivo* observations, CLL cells within the scaffold downregulated the chemokine receptor CXCR4, further reduced upon proliferative stimulation. Final validation involved treatment with targeted therapies: the BCL-2 antagonist venetoclax and the BTK inhibitor ibrutinib. Venetoclax treatment revealed greater CLL protection within the LN environment than in BM, whereas the mobilizing effect of ibrutinib was comparable between these two niches. This 3D LN model offers an effective *ex vivo* platform for studying microenvironment–tumour interactions and tissue-specific drug responses.

## Teaser

**Summary:** We developed a 3D tissue-like lymph node model recapitulating the malignant microenvironment of chronic lymphocytic leukaemia, enabling investigation of disease evolution and therapeutic response driven by microenvironmental interactions.

## INTRODUCTION

Chronic lymphocytic leukaemia (CLL) is characterized by the progressive expansion and accumulation of CD5^+^ B cells that recirculate in the peripheral blood (PB) and accumulate in the bone marrow (BM) and secondary lymphoid organs (Caligaris-Cappio and Hamblin, 1999; Scarfò et al., 2016). Lymph nodes (LNs) are considered key reservoirs of the disease, as CLL cells tend to organize in proliferation centres (PCs) (Herishanu et al., 2011a), in which large, actively dividing leukaemic cells expand and fuel disease progression in the periphery (Herndon et al., 2017). The proliferating leukaemic fraction residing in these tissues likely represents the primary source of relapse in patients with CLL (Hallek, 2025). Despite major advances in therapy, including BTK inhibitors and BCL-2 antagonists, CLL remains incurable (Burger and O'Brien, 2018). These therapies often show reduced efficacy against CLL cells within supportive microenvironments in the BM and especially in the LNs. In these niches, leukaemic cells acquire treatment resistance through exposure to soluble factors released by bystander cells, direct cell–cell contact (a mechanism known as cell adhesion-mediated drug resistance; Kim et al., 2022), or adhesion to the extracellular matrix (ECM) (Caligaris-Cappio et al., 2014; Anderson and Simon, 2020). The interactions that CLL cells establish with the surrounding tissues are, thus, critical in promoting their survival, proliferation and resistance to therapy. Notably, understanding how different tissue microenvironments influence leukaemic behaviour and drug response is, therefore, essential (Burger, 2011). CLL cells adopt distinct phenotypes depending on their tissue localization during recirculation. For example, the fraction of CLL cells that has recently exited tissue compartments can be identified in the circulation as CXCR4^low^ (Pasikowska et al., 2016). In the PB, CLL cells remain quiescent, whereas in the BM, they primarily receive protective and pro-survival signals from surrounding cells (Burger, 2011; Dubois et al., 2020; Ten Hacken and Burger, 2014; Herishanu et al., 2013). In the LNs, leukaemic cells are exposed to signals that promote proliferation and activation in the PCs (Herndon et al., 2017; Pasikowska et al., 2016), including the stimulation of the B-cell receptor and NFκB pathways, among others (Ntoufa et al., 2016; Mazzarello et al., 2023). These signals originate from surrounding cells (Ten Hacken and Burger, 2014; Kipps et al., 2017), including T cells, nurse-like cells (NLCs), follicular dendritic cells, fibroblastic reticular cells and endothelial cells, as well as from ECM components such as fibronectin (FN), collagen and hyaluronic acid (HA) (Girbl et al., 2013). Soluble factors also contribute to this supportive niche (Dubois et al., 2020). Within these structures, CLL cells engage in homotypic interactions with each other and heterotypic interactions with stromal and immune cells (Burger et al., 2009; Minici et al., 2017). Given the central role of the LN microenvironment in CLL pathogenesis, progression and therapy resistance, there is a clear need for *in vitro* models that accurately recapitulate this niche. Ideal models should reflect the cellular diversity of the LN, support homotypic and heterotypic interactions, and reproduce its three-dimensional (3D) architecture (Scielzo and Ghia, 2020; Barozzi and Scielzo, 2023). To date, most CLL *in vitro* models have been limited to scaffold-free constructs, lacking the microenvironmental and structural complexity, as well as the possibility to study leukaemic cells trafficking inside and outside the 3D tissue. To address this technology gap, we adapted our previously validated protocol for a 3D BM surrogate (Barbaglio et al., 2021; Belloni et al., 2022, 2018) to generate a 3D dynamic LN model within a clinorotator bioreactor, the Rotary Cell Culture System (RCCS^TM^). The aim was to more closely recapitulate the CLL LN microenvironment, mimicking the complex interactions and the dynamics found *in vivo*.

## RESULTS

### LN stromal cells support CLL viability in 3D culture

**Fig. 1.**
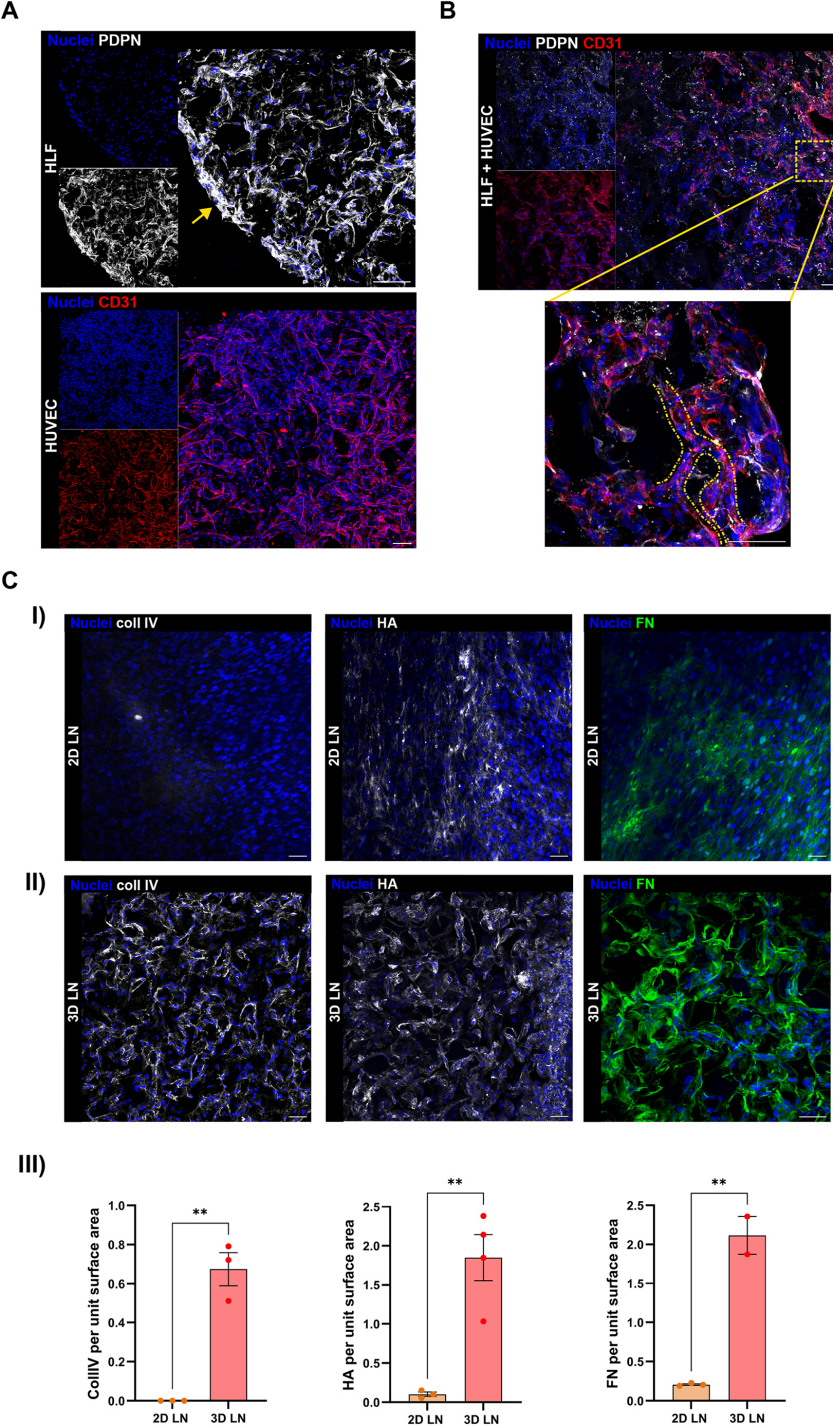
**Immunofluorescence confocal reconstructions of 3D mono- and co-cultures.** (A) Top: 3D mono-culture of human lymphatic fibroblasts (HLFs) organized into structures expressing podoplanin (PDPN). The yellow arrow indicates the capsule-like structure produced by fibroblasts within the scaffold. Scale bar: 100 µm. Bottom: 3D mono-culture of human umbilical vein endothelial cells (HUVECs) forming structures positive for CD31. Scale bar: 100 µm. (B) 3D co-culture of HLFs and HUVECs expressing CD31 and PDPN. Yellow dashed lines indicate tubular-like structures formed by endothelial cells. Scale bars: 50 µm. This experiment was repeated three times in the laboratory. These images derive from one representative sample per condition. (C) I: 2D LN co-culture cells produce extracellular matrix (ECM) components including collagen IV (coll IV), fibronectin (FN) and hyaluronic acid (HA). Scale bar: 50 µm. II: 3D LN co-culture cells produce ECM components including coll IV, FN and HA. III: image quantification of the surface (2D) and volume (3D) occupied by the ECM protein (i.e. coll IV, HA, FN), normalized to the imaged surface area (coll IV, *n*=3, unpaired two-tailed *t*-test, **P=0.0013; HA, 2D *n*=3, 3D *n*=4, unpaired two-tailed *t*-test, **P=0.0040; FN, 2D *n*=3, 3D *n*=2, unpaired two-tailed *t*-test, **P=0.0018). The samples were collected from the bioreactor and processed as described in the Materials and Methods section.

Given the critical role of the LN microenvironment in promoting CLL expansion and drug resistance, we aimed to generate a 3D model that replicates the interactions between leukaemic cells and the LN niche for testing both approved and novel therapies. To achieve this, we adapted our previously validated protocol for reconstructing the BM microenvironment in 3D culture (Belloni et al., 2022) by using specific LN stromal cells, thus tailoring the culture conditions to their need (i.e. medium type and culture time). We first defined the optimal conditions for establishing the 3D LN model, starting with the selection of cellular components (Fig. S1). Consistent with previous reports (Burger et al., 2000; Roessner and Seiffert, 2020), CLL-peripheral blood mononuclear cells (PBMCs) maintained higher viability than purified CLL cells (CLL-PUR) over 5 days in two-dimensional (2D) suspension cultures, likely owing to the presence of other immune components (e.g. T cells and macrophages) that provide additional survival support (Fig. S1A). We therefore selected CLL-PBMCs for model generation, allowing for enrichment with the patient's native immune microenvironment. To set the best experimental conditions of the cell cultures for generating our 3D model, we first performed 2D experiments evaluating CLL cell viability in relation to the other cell components and medium. At first, we evaluated 2D co-cultures of both CLL-PBMCs and CLL-PUR with human umbilical vein endothelial cells (HUVECs; as endothelial mimicking) or human lymphatic fibroblasts (HLFs; as fibroblastic reticular LN stromal cells) expressing vascular cell adhesion molecule-1 (VCAM-1), the ligand for VLA-4, which is essential for CLL survival (Dal Bo et al., 2014) (Fig. S1B). Both HLFs and HUVECs well supported the viability of CLL-PUR and CLL-PBMCs in 2D co-culture up to 5 days (Fig. S1C-I,II). Moreover, CLL-PBMCs co-cultured in 2D conditions with HLFs+HUVECs (referred to hereafter as LN stroma) showed a higher viability, if compared with their basal 2D suspension mono-culture after 5 days (Fig. S1D). We then proceeded to establish the 3D cultures. We first tested the ability of the LN stroma to populate the Spongostan^TM^ gelatine scaffold by seeding HLFs and HUVECs separately and then in combination. In this way, we were able to verify the intrinsic ability of each cell type to adhere to and populate the gelatine matrix while maintaining specific functional markers: podoplanin (PDPN) for HLFs (Roet et al., 2024) and CD31 (also known as PECAM1) for HUVECs (Newman, 1997) (Fig. 1A). Cells were then seeded together at a 1:1 or 1:2 ratio (HLFs: HUVECs), depending on HUVEC proliferation rates, which varied across experimental batches. After 5 days, HLFs and HUVECs efficiently colonized the scaffold and, again, retained their respective lineage markers (Fig. 1B; Fig. S2C). HLFs alone, as well as in the 3D co-culture, formed a dense fibroblast network throughout the scaffold (Fig. 1A,B) and generated a capsule-like structure at the periphery, reminiscent of LN architecture (Fig. 1A, yellow arrow). Moreover, HUVECs, supported by HLFs, formed capillary-like structures, as shown in Fig. 1B by the yellow dashed lines. We next examined ECM deposition, which is crucial for maintaining tissue integrity and supporting CLL cell activation and survival (Dal Bo et al., 2014; Herishanu et al., 2011a), as well as influencing tissue composition and stiffness (Sampietro et al., 2025), in 2D and 3D co-cultures after 5 days (Fig. 1C). Primary antibody reactivity with the scaffold material was evaluated to exclude potential affinity with the matrix itself (Fig. S2A), which was highlighted in the images in Fig. S2A by exploiting its slight autofluorescence. The stroma in both conditions actively produced and deposited ECM components, yet with differences among the two conditions. Stromal and endothelial cells cultured in 2D conditions (Fig. 1C-I) showed no ability to produce collagen IV (Zhou et al., 2016), which was clearly produced and deposited by the same cells in 3D scaffolds (Fig. 1C-II). HA (Girbl et al., 2013) and FN (Patten and Wang, 2021) were observed to be produced in both conditions. Quantification of the deposited matrix revealed a significant increase in ECM protein production by the same cells in the 3D condition, emphasizing the role of the three-dimensionality and dynamicity of the 3D culture in stimulating the generation of a more *in vivo*-like structure (Fig. 1C-III). Notably, organized collagen IV fibres were observed only in the 3D LN co-cultures and not in mono-cultures of HLFs or HUVECs (Fig. S2B). These findings underscore the necessity of stromal–endothelial cooperation and crosstalk for specific ECM production (Zhou et al., 2016), indicating a complex and coordinated interaction between the two cell types within the 3D construct. Moreover, the ECM production and structural organization in the co-culture strengthens the biological relevance of our 3D LN model for future functional studies.

### CLL-PBMCs populate the LN scaffold, retaining high viability

**Fig. 2.**
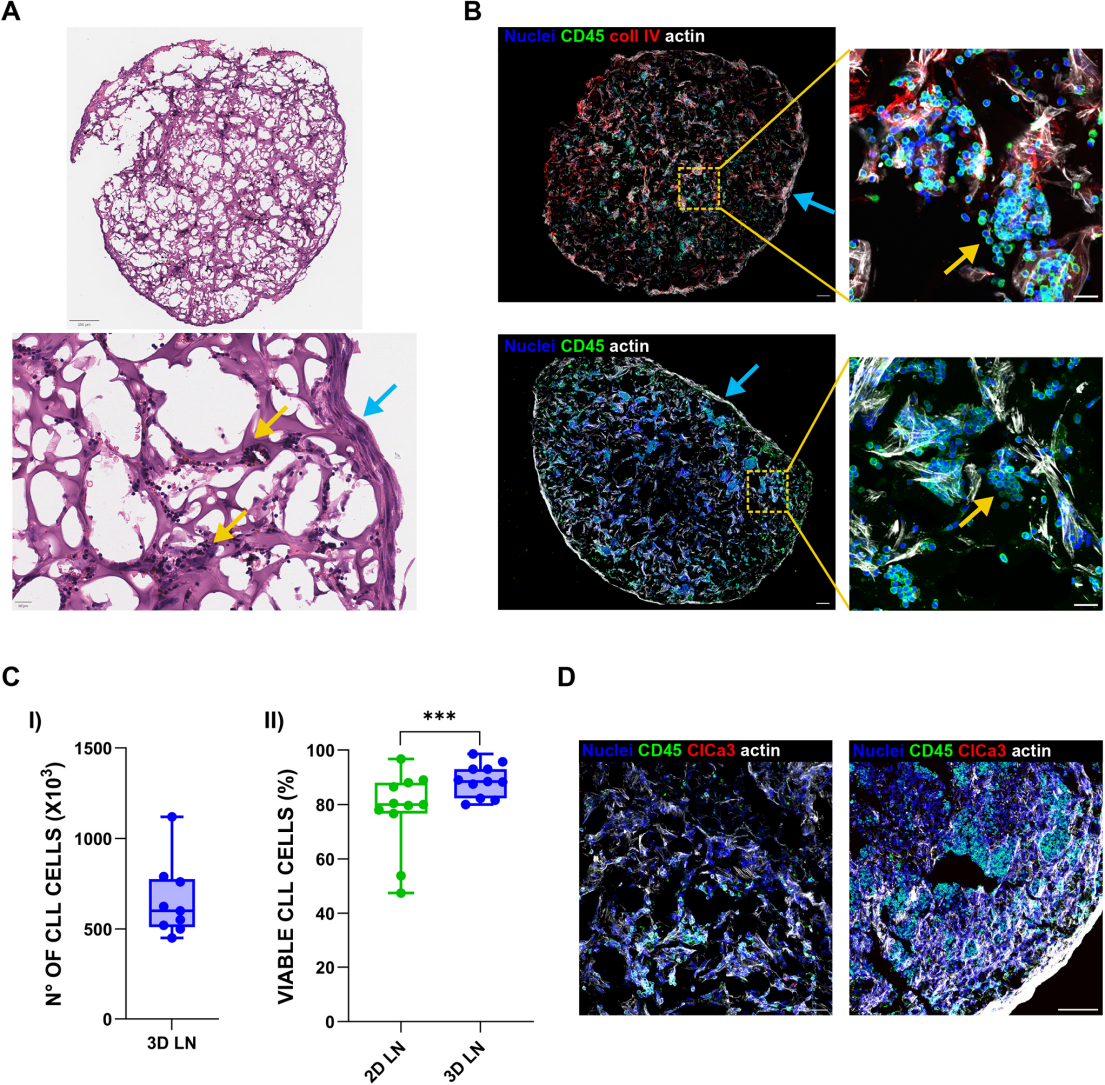
**Chronic lymphocytic leukaemia (CLL) cells populate the scaffold and maintain high viability.** (A) Haematoxylin and Eosin staining of CLL-peripheral blood mononuclear cells (PBMCs) within the LN scaffold. Top: low-magnification view showing the entire scaffold diameter. Scale bar: 250 μm. Bottom: higher-magnification image showing CLL cells forming clusters (yellow arrows) and LN stromal cells generating a capsule-like structure (light-blue arrow). Scale bar: 20 μm. (B) Immunofluorescence imaging of the LN scaffold populated by CLL cells. Left column: 3D reconstruction of a scaffold section showing the full diameter and a peripheral LN-like capsule (light-blue arrows). Scale bars: 100 μm. Right column: magnified views of the same samples. Yellow arrows indicate clusters of CLL cells. Scale bars: 20 μm. (C) I: quantification of CLL cells recovered from 3D LN scaffolds by Trypan Blue exclusion dye after enzymatic digestion. Values were normalized to the percentage of CD19^+^CD5^+^ cells measured by flow cytometry (*n*=9). II: viability of CLL-PBMCs after 5 days in 2D versus 3D LN co-cultures (*n*=11, Wilcoxon signed-rank test for paired samples, ***P=0.0010). In box-and-whisker plots, the middle line represents the median, the box represents the interquartile range (25th–75th percentiles), and the whiskers represent 1.5 times the interquartile range. Individual points outside the whiskers represent biological outliers. (D) Immunofluorescence images of long-term (15-day) 3D LN cultures from two different CLL patient samples. Scale bars: 100 μm.

**Fig. 3.**
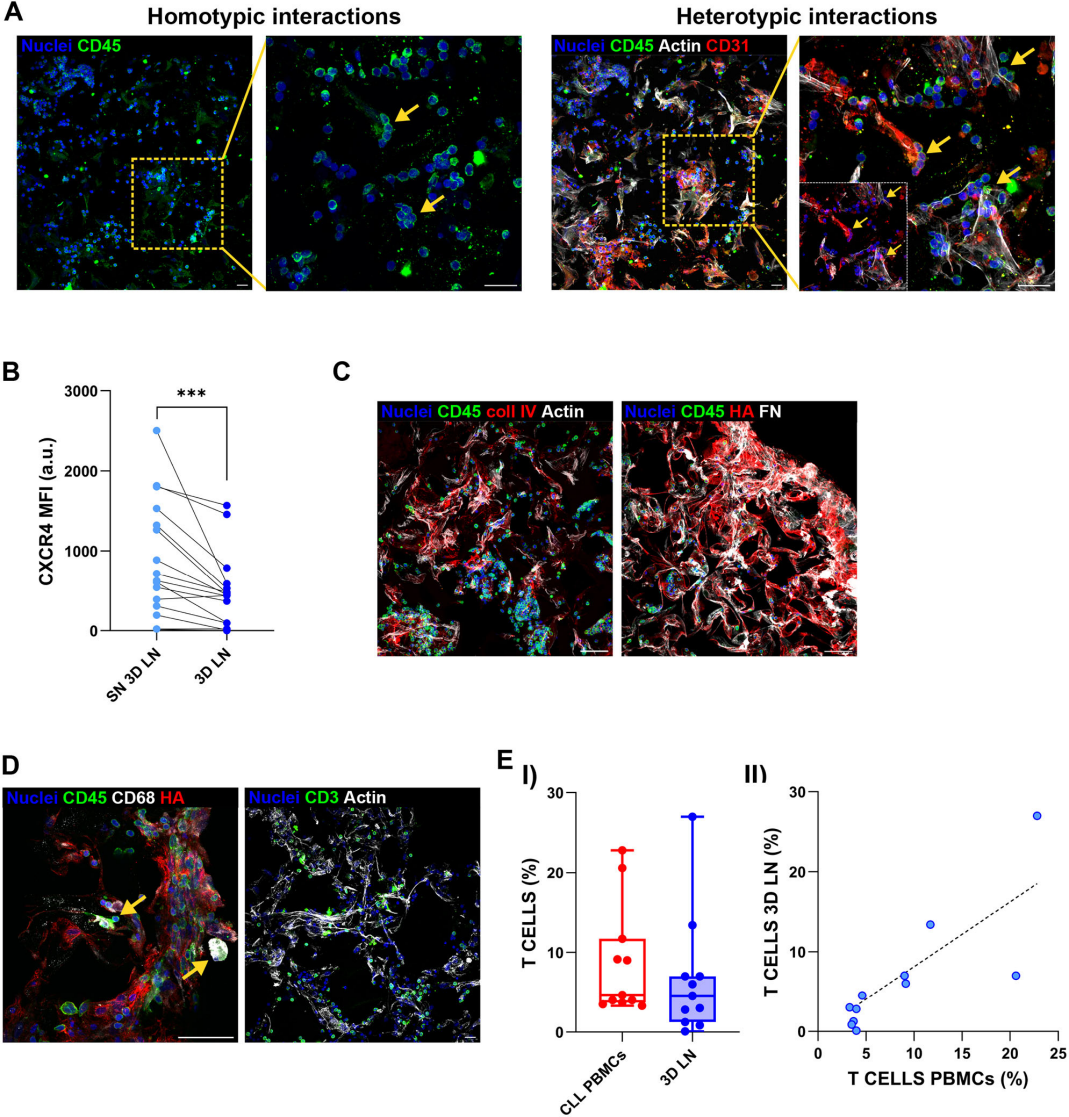
**CLL cells interact with the microenvironment.** (A) Immunofluorescence 3D reconstructions of the LN scaffold populated with CLL-PBMCs, showing homotypic interactions between CLL-PBMCs (left) and heterotypic interactions between CLL-PBMCs, stromal cells and ECM components (right). Yellow arrows indicate homotypic and heterotypic interactions. Scale bars: 20 µm. (B) Flow cytometric analysis of CXCR4 mean fluorescence intensity (MFI) in CD19^+^CD5^+^ cells (*n*=16, Wilcoxon signed-rank test for paired samples, ***P=0.0002). a.u., arbitrary units. (C) Immunofluorescence 3D reconstructions showing deposition of ECM proteins in LN scaffolds populated with CLL-PBMCs. Scale bars: 200 µm (left); 50 µm (right). (D) Immunofluorescence 3D reconstructions showing the presence of additional immune cell populations within the LN scaffold populated with CLL-PBMCs. Yellow arrows indicate CD68^+^ cells. (E) Flow cytometry analysis of T cells within the scaffold. I: percentage of T cells inside the scaffold (*n*=11). In box-and-whisker plots, the middle line represents the median, the box represents the interquartile range (25th–75th percentiles), and the whiskers represent 1.5 times the interquartile range. Individual points outside the whiskers represent biological outliers. II: correlation between the percentage of T cells in the scaffold and the initial percentage in PBMCs [*n*=11; correlation was analysed using Pearson's *r* (*P*=0.0019, *R*^2=^0.6746, *r=*0.8214); a simple linear regression line was fitted to the data to visualize the trend].

After confirming that the LN stroma efficiently populates the scaffolds, to evaluate whether CLL cells could successfully infiltrate and survive in the LN-like environment, we added PBMCs from patients with CLL to the culture. After 5 days, we characterized the resulting CLL LN constructs by Haematoxylin and Eosin staining, immunofluorescence and cell counting using Trypan Blue exclusion dye. CLL-PBMCs successfully entered and uniformly populated the scaffold, forming clusters among stromal cells (Fig. 2A,B, yellow arrows), which, in turn, generated a LN-like capsule structure at the scaffold periphery (Fig. 2A,B, light-blue arrows). To evaluate the impact of the 3D model on CLL viability, we compared 3D co-cultures to matched 2D co-cultures. Viability was assessed in CD19^+^CD5^+^ cells using Annexin V/propidium iodide (PI) or fixable viability dye staining. Although direct comparison between 2D and 3D cultures are limited by differences in stromal and endothelial cell growth rates and morphological behaviour across the two systems (Barozzi and Scielzo, 2023), CLL-PBMCs homed within the 3D LN scaffold for up to 5 days (Fig. 2C-I) and exhibited significantly overall higher viability than cells in the 2D condition (Fig. 2C-II). To highlight the potentialities of these 3D CLL LN cultures, we performed longer-term experiments (i.e. 15 days), not possible in two dimensions, demonstrating that, in our model, CLL cell viability can be preserved, as observed by minimal cleaved caspase 3 (ClCas3) signal in CD45^+^ cells. These findings suggest that the stromal context and 3D architecture provided survival hints that prevented the early cell death, commonly seen in primary CLL samples cultured without external stimuli (Fig. 2D). Immunofluorescence confirmed that CLL cells were evenly distributed throughout the scaffold and capable of forming both homotypic (Fig. 3A, left, yellow arrows) and heterotypic (Fig. 3A, right, yellow arrows) interactions with stromal cells and the ECM. To assess whether the 3D model recapitulates *in vivo* tissue-like behaviour, we evaluated the expression of CXCR4, a chemokine receptor typically downregulated in LN-resident CLL cells. Indeed, we confirmed the downregulation of CXCR4 in CLL cells located inside the LN scaffold compared to cells floating in the surrounding medium (Fig. 3B) (Pasikowska et al., 2016; Calissano et al., 2011; Friedman et al., 2024). We also examined the presence and distribution of ECM components in the presence of CLL cells. Deposition of collagen IV, FN and HA was confirmed throughout the scaffold (Fig. 3C). As shown in Fig. S2D, in our published 3D BM microenvironment (Barbaglio et al., 2021), we did not detect production and further deposition of HA, which was, by contrast, present in the 3D LN scaffolds. Finally, given the established roles of T cells, macrophages and NLCs in supporting CLL survival and proliferation (Burger et al., 2000; Roessner and Seiffert, 2020; Galletti et al., 2016), we assessed whether these accessory cells could effectively home to the scaffold. Immunofluorescence analysis confirmed their presence across all patient samples analysed (Fig. 3D). Flow cytometry analysis further quantified T-cell percentage within the 3D LN, which was proportional to their original abundance in the PBMC population prior to seeding (Fig. 3E-I,II). The effective homing and proportional representation of T cells (and presence of other accessory cells) support the physiological fidelity of the model and its ability to reflect the native tumour microenvironment.

### CpG and IL-2 induce CLL cell proliferation inside the scaffold

**Fig. 4.**
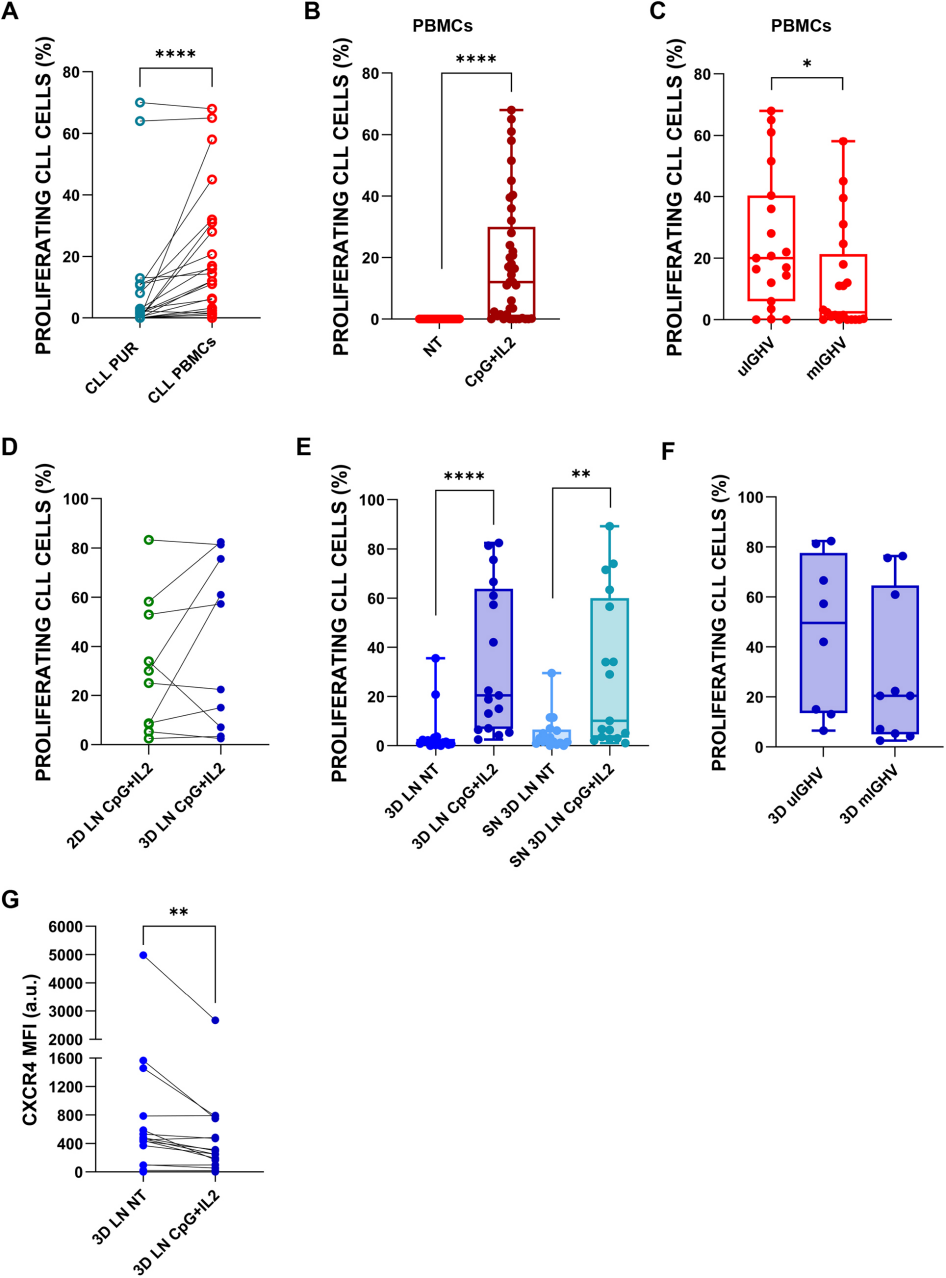
**CLL cell proliferation.** Flow cytometry analysis of CLL cells under various culture conditions: (A) Comparison of proliferation between CLL-PBMCs and purified CLL cells (CLL-PUR) following CpG+IL2 stimulation (*n*=25, Wilcoxon signed-rank test for paired samples, ****P<0.0001). (B) Proliferation of CLL-PBMCs cultured with or without CpG and IL-2 (*n*=23, Wilcoxon signed-rank test for paired samples, ****P<0.0001). (C) Comparison of CpG+IL2-induced proliferation between unmutated (uIGHV) and mutated (mIGHV) CLL cells (uIGHV *n*=19, mIGHV *n*=21, Mann–Whitney *U* test, *P=0.0270). IGHV, immunoglobulin heavy chain variable region. (D) Paired comparison of CLL cell proliferation among 2D and 3D conditions in the presence of proliferation stimuli (*n*=10). (E) Proliferation of CLL-PBMCs in 3D culture, both inside the scaffold and in the supernatant [*n*=17, Friedman test with Dunn's multiple comparisons test; 3D LN not treated (NT) versus 3D LN CpG+IL2, ****P<0.0001; 3D SN NT versus 3D SN CpG+IL2, **P=0.0026]. (F) Comparison of uIGHV and mIGHV CLL cell proliferation in 3D cultures following CpG+IL2 stimulation (uIGHV *n*=8, mIGHV *n*=10). (G) Flow cytometric evaluation of CXCR4 MFI in CLL cells after CpG+IL2 stimulation (*n*=16, Wilcoxon signed-rank test for paired samples, **P=0.017). In box-and-whisker plots, the middle line represents the median, the box represents the interquartile range (25th–75th percentiles), and the whiskers represent 1.5 times the interquartile range. Individual points outside the whiskers represent biological outliers.

To recapitulate key proliferative signals of the LN environment, we added CpG-oligodeoxynucleotide (ODN) (Toll-like receptor 9 agonist) (1 µg/ml) and IL-2 (T cell-derived cytokine) (500 U/ml) to the cultures, based on previous evidence showing that this combination most effectively induces proliferation in leukaemic cells (Primo et al., 2018). Before applying this protocol in the 3D LN model, we evaluated its efficacy in 2D cultures of CLL-PBMCs and CLL-PUR to confirm the contribution of other immune cell types in the presence of external stimuli (Haselager et al., 2023; Galletti et al., 2016). We stained the cells with CellTrace Violet™ or Vybrant™ CFDA-SE and analysed them after 5 days. Proliferation was more robust in CLL-PBMCs than in CLL-PUR cultured in two dimensions (Fig. 4A). Consistently, we also observed higher proliferation in CLL-PBMCs stimulated with proliferative stimuli than in their non-stimulated counterparts (Fig. 4B; Fig. S3A), confirming the supportive role of additional cellular components in the PBMC fraction as well as in the effect of CpG-ODN+IL-2. Notably, CLL-PBMCs from patients with progressive disease and/or unmutated immunoglobulin heavy chain variable region (IGHV) (uIGHV), which are features associated with poorer prognosis, exhibited significantly higher proliferation than those with mutated IGHV (mIGHV), typically linked to indolent disease (ten Hacken et al., 2019) (Fig. 4C). These findings highlight inter-patient variability in proliferative potential and suggest an intrinsic difference in CLL cell responsiveness to stimulation. We then assessed the proliferative response of CLL cells in the presence of 2D and 3D LN stroma. A direct comparison of proliferation between 2D and 3D co-cultures did not reveal a statistically significant difference (Fig. 4D); however, clear increases or reductions in proliferation were observed across individual samples, indicating that our 3D model preserves the *in vivo* heterogeneity of patients' cells (Fig. S3B). Moreover, CpG-ODN+IL-2 cocktail stimulated the proliferation of CLL cells inside the scaffold as well as those floating in the supernatant (Fig. 4E). Subgroup analysis revealed, again, a trend toward higher proliferation in uIGHV cases (Fig. 4F), although the difference was less pronounced in 3D than in 2D mono-cultures, possibly due to the influence of stromal interactions on CLL. Finally, as CXCR4 downregulation was observed in leukaemic cells once they had entered in the 3D LN constructs (Fig. 3B), we investigated the effects of CpG-ODN and IL-2 on CXCR4 expression within the scaffold. This stimulation further reduced CXCR4 levels in CLL cells cultured in the 3D LN environment (Fig. 4G; Fig. S3C), consistent with *in vivo* patterns (Pasikowska et al., 2016). Because CXCR4 downregulation reflects the LN-resident CLL phenotype, our data support the physiological fidelity of the 3D LN scaffold.

### CLL-PBMC differential response to targeted therapies in the 3D LN and BM models

**Fig. 5.**
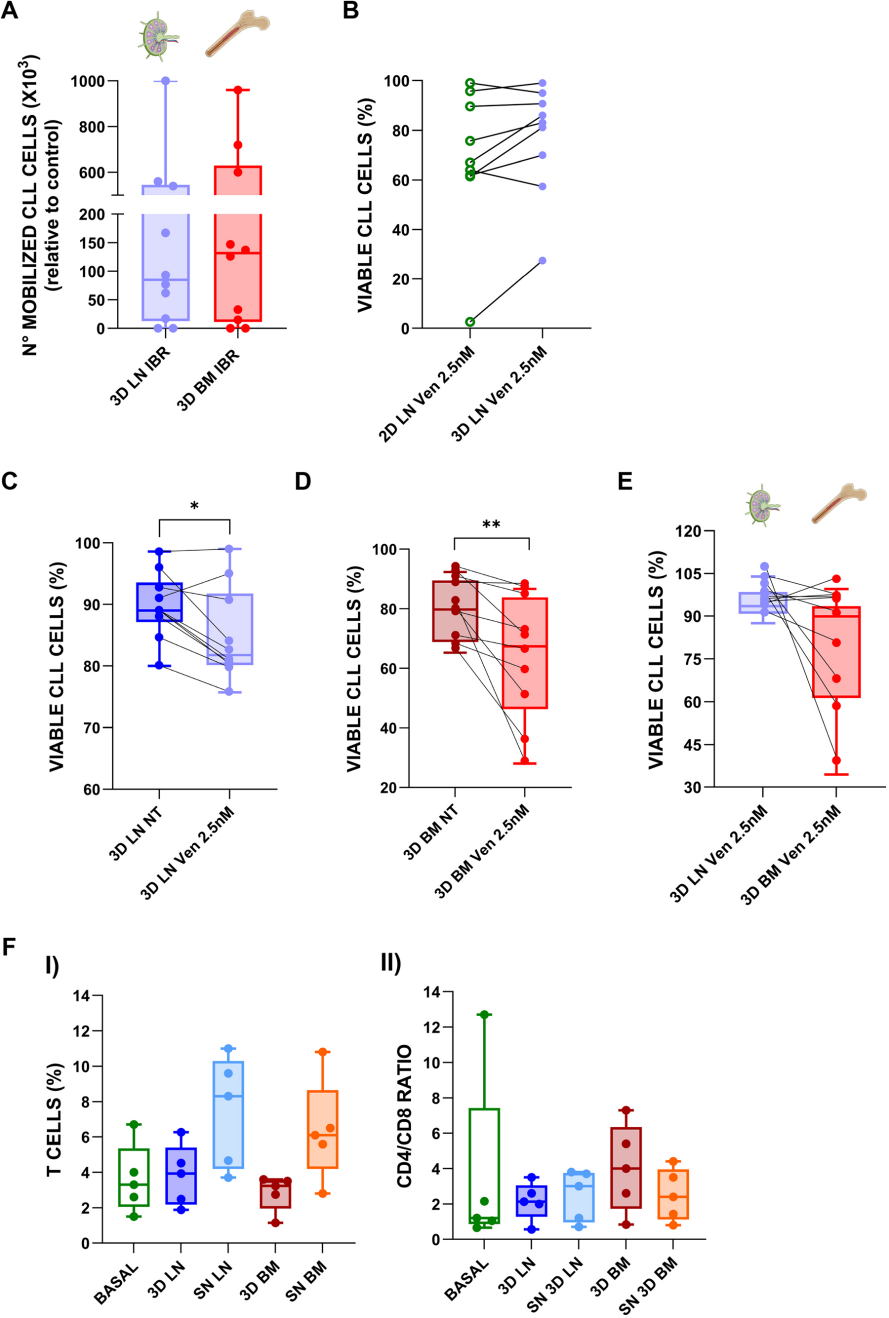
**Effects of ibrutinib and venetoclax on 3D LN and bone marrow (BM) CLL co-cultures.** Flow cytometry analysis of CLL cells in different experimental conditions. (A) CLL cell mobilization following treatment with 1 µM ibrutinib (ibr). Mobilized cells were quantified in the supernatant (SN) of the LN and BM scaffolds (*n*=10). (B) 2D versus 3D LN response to 2.5 nM venetoclax (ven) (*n*=9) (C) 3D LN co-cultures NT and treated with 2.5 nM ven (*n*=10, Wilcoxon signed-rank test for paired samples, *P=0.0137). (D) 3D BM co-cultures NT and treated with 2.5 nM ven (*n*=10, Wilcoxon signed-rank test for paired samples, **P=0.0020). (E) Comparison among 3D NT LN and BM co-cultures normalized to 3D LN and BM co-cultures treated with 2.5 nM ven (*n*=10, paired two-tailed *t*-test, *P*=0.0741). (F) I: percentage of T cells in basal patient blood samples and in the corresponding CLL-PBMCs cultured in 3D LN and BM co-cultures, as well as in the respective SNs (*n*=5). II: CD8/CD4 ratio in the same conditions (*n*=5). In box-and-whisker plots, the middle line represents the median, the box represents the interquartile range (25th–75th percentiles), and the whiskers represent 1.5 times the interquartile range. Individual points outside the whiskers represent biological outliers.

To determine whether the 3D LN model could be used to evaluate drug responses, we compared its behaviour to that of the previously validated 3D BM model (Barbaglio et al., 2021), focusing on two clinically relevant agents: the BCL-2 antagonist venetoclax (ven) (Anderson et al., 2024) and the BTK inhibitor ibrutinib (ibr) (Chen and Chiorazzi, 2023). 3D culture conditions in terms of timing and cell number are comparable among LN and BM models. The two microenvironments differ in stromal cell types, medium (i.e. presence of endothelial medium in the LN model) and ECM content. The BM microenvironment has already been evaluated for drug testing (Barbaglio et al., 2021) for the LN scaffold, permeability was tested using dextran-FITC (70 kDa), which exceeds the molecular mass of both ven (868.4 Da) and ibr (440.5 Da), validating diffusion capacity through the matrix (Fig. S4). For the LN system, a modified version of the BM protocol was used (Belloni et al., 2022). We first tested the mobilizing effect of ibr in both models. CLL-PBMCs seeded in the LN scaffold were treated with ibr, and, in paired experiments, the drug induced a comparable level of mobilization in both the LN and BM models, mirroring what happens *in vivo* (Herman et al., 2014) (Fig. 5A). We then proceeded to assess the effects of ven by first treating CLL-PBMCs in 2D mono-culture with three different concentrations (2.5, 10 and 20 nM) for 48 h. In this patient cohort, ven induced significant cell death at the lowest concentration of 2.5 nM (Fig. S5A). We therefore selected this dose for subsequent experiments in 2D and 3D co-cultures. The comparison of 2D and 3D LN co-cultures treated with ven 2.5 nM displayed no overall differences in CLL cell viability, highlighting the heterogeneity among primary samples also in the context of therapy outcome (Fig. 5B). To investigate how different microenvironments influence ven efficacy, we compared the response of CLL cells cultured in BM and LN scaffolds. Both models displayed high baseline viability; however, BM scaffolds showed a slightly lower mean viability than that of LN scaffolds (79.76% versus 89.79%, respectively). When comparing absolute viability after treatment (Fig. 5C,D), the BM environment showed a significantly higher response than that of the 3D LN to ven. To decouple the drug's specific effect from baseline differences, we also analysed the data, by normalizing each treated sample to its NT control (Fig. 5E). Although this normalization led to a loss of formal statistical significance, a consistent trend (*P*=0.0741) toward higher sensitivity in the BM environment remained (mean normalized viability, 78.77% in BM versus 94.49% in LN). Of particular interest was the pronounced heterogeneity of response within the BM, in which viability values were more widely dispersed (28-86.60%; 20.5 s.d.) than in the LN model (75.7-99%; 7.36 s.d.). This observation suggests that different lymphoid microenvironments can uniquely influence CLL cell behaviour and response to drugs. Additional experiments using higher ven concentrations (10 and 50 nM) confirmed a trend for higher protection in the LN model; however, these doses exerted such strong overall toxicity that the differential protection effect and statistical significance were less evident (Fig. S5B). To investigate whether T cells contributed to the potential ven resistance in the LN scaffold, as previously suggested *in vitro* (Elias et al., 2022), we compared T-cell abundance, subset distribution and CD4/CD8 ratios in the 3D LN and BM co-cultures. No major differences were observed in the overall percentage of T cells (Fig. 5F-I,II; Fig. S5C), apart from a slight decrease in the CD4/CD8 ratio in the 3D LN (Fig. 5F-II). These results indicate that T cells are unlikely to drive ven resistance in this model system. These data reinforce our model's potential for testing drug responses in a physiologically relevant context. Indeed, ibr exerts a mobilizing effect in both LN and BM models (Herman et al., 2014). Interestingly, ven treatment displayed a trend for increased sensitivity of CLL cells in the 3D BM microenvironment over the 3D LN counterpart, reflecting *in vivo* observations (Zielonka and Jamroziak, 2024; Skanland and Mato, 2021) and, notably, a more patient-specific response profile.

## DISCUSSION

CLL cells critically depend on signals from the tissue microenvironment, particularly the BM and LN, to evade apoptosis, sustain proliferation and acquire resistance to therapy. These protective processes are mediated by complex interactions with stromal and endothelial cells, immune bystanders, soluble factors such as chemokines and cytokines, and components of the ECM (Caligaris-Cappio et al., 2014; Anderson and Simon, 2020; Burger, 2011; Dubois et al., 2020; Ten Hacken and Burger, 2014). Although the protective role of the microenvironment is well recognized, the relative contributions of different tissue compartments, and how these shape CLL behaviour, remain incompletely understood. Given the central role of the LN in sustaining leukaemic proliferation and generating drug-resistant populations (Herndon et al., 2017; Pasikowska et al., 2016), *in vitro* models that accurately mimic LN-specific microenvironmental features are essential for studying CLL pathophysiology and therapeutic response. Traditional 2D cultures, although widely used, do not capture the spatial organization or signalling complexity of tissue microenvironments. By contrast, 3D culture systems restore physiological cell morphology, polarity and intercellular interactions, and potentially more accurately reflect *in vivo* drug responses (Barozzi and Scielzo, 2023). The available murine models (Bertilaccio and Chen, 2024), although useful, often fail to recapitulate key aspects of human CLL biology, further underscoring the need for physiologically relevant preclinical models. To recapitulate LN-specific features, support CLL viability and proliferation, and assess tissue-specific drug responses, here, we developed a 3D LN surrogate model using an RCCS^TM^ bioreactor and compared it with our previously established 3D BM model (Barbaglio et al., 2021; Belloni et al., 2022). Scaffold design was a key element to support viability, cell organization and mechanical support (Barozzi et al., 2025). To meet this need, a gelatine-based matrix was selected for its high biocompatibility, as previously validated in the BM setting. To reproduce LN architecture, we incorporated HLFs as stromal cells and HUVECs as endothelial cells. HLFs express VCAM-1, which engages VLA-4 on CLL cells and promotes survival signalling pathways (Dal Bo et al., 2014), while HUVECs support CLL proliferation through CD31–CD38 interactions (Deaglio et al., 2010; Brachtl et al., 2014). We selected this scaffold because it supports ECM deposition, including HA (Girbl et al., 2013), collagen IV (Bahramsoltani et al., 2014) and FN (Patten and Wang, 2021; De et al., 1999), components that provide mechanical and biochemical cues essential to the LN niche (Herishanu et al., 2013; Kramer et al., 1988; Ponzoni et al., 2011). Indeed, we showed that our 3D LN scaffold sustained CLL cell viability and proliferation (preserving the high inter- and intra-patient variability), particularly following stimulation with CpG and IL-2 in both 2D and 3D co-cultures (Fig. 4). Despite inter-patient variability also observed *in vivo*, the 2D experiments confirmed that CLL-PBMCs proliferated more effectively than CLL-PUR, highlighting the supportive role of bystander immune cells. Notably, PBMCs from patients with progressive disease also showed a trend for higher proliferation, possibly reflecting intrinsic differences in proliferative potential and anergy (Caligaris-Cappio, 2014; Haselager et al., 2023; ten Hacken et al., 2019). Compared to other 3D LN CLL models, which lack a complex 3D stromal architecture and endothelial compartment (Haselager et al., 2023; Lenti et al., 2025), our scaffold ensures uniform stromal distribution and a structured, reproducible architecture that accommodates key immune populations such as T cells (CD4^+^ and CD8^+^) and macrophages. The dynamic bioreactor setup allows for compartmentalized analysis of cells inside and outside the scaffold, enabling the study of CLL mobilization (Barbaglio et al., 2021). Importantly, the system recapitulates *in vivo* trafficking patterns: CLL cells residing in the scaffold display reduced CXCR4 expression compared to those remaining in suspension, mirroring LN–PB differences observed in patients (Pasikowska et al., 2016). Given the role of the microenvironment in drug resistance (Skanland and Mato, 2021), we evaluated the effects of ven and ibr in both LN and BM 3D models. These targeted therapies represent two categories of drugs widely used in clinic, with two distinct mechanisms of action and microenvironmental sensitivity. The LN scaffold conferred greater resistance to ven than 2D co-cultures. Moreover, when comparing the two 3D models, the LN microenvironment provided more protection from ven than the BM, consistent with *in vivo* data (Anderson et al., 2016). T-cell content did not account for these differences, suggesting that other factors could be involved. A possible explanation for the increased ven resistance in the LN model could be related to differences in ECM composition. In particular, we observed higher production of HA in the LN scaffold compared with the BM one (Fig. 3; Fig. S2D). HA is the main ligand of CD44, a receptor highly expressed by CLL cells (Girbl et al,. 2013), and its engagement has been reported to promote leukaemic cell activation and survival (Herishanu et al., 2011b). Interaction between HA and CD44 can trigger pro-survival signalling pathways, ultimately leading to the upregulation of anti-apoptotic proteins such as MCL-1 (Herishanu et al., 2011b; Haselager et al., 2020; Takács et al., 2021; D'Arena et al., 2014). Our model could thus serve as a platform to explore this mechanism further, particularly in combination with agents that target this pathway as reported in literature for acute myeloid leukaemia (Yu et al., 2020). Interestingly, drug-induced CLL mobilization was comparably reproduced in both BM and LN models, consistent with known *in vivo* pharmacodynamics (Herman et al., 2014). These overall data on drug response of CLL cells in our 3D LN and BM microenvironments suggest plasticity of the models to study different therapies and, eventually, their combination (e.g. ibr+ven) following the protocols of multiple administrations (Eichhorst et al., 2023; Kater et al., 2022).

In summary, our 3D LN model offers a physiologically relevant platform for studying CLL biology, microenvironmental crosstalk and tissue-specific drug responses. Although it is not designed for high-throughput screening, it provides a robust tool to dissect mechanisms of microenvironment-mediated drug resistance, compare responses across tissue compartments and inform predictive preclinical testing. Its ability to integrate immune cells, stromal support and ECM architecture makes it particularly well suited to investigate the interplay between CLL cells and their niche, and to evaluate therapies targeting tissue-specific vulnerabilities.

## MATERIALS AND METHODS

### Ethical approval and study population

Patients with CLL were diagnosed according to the 2018 International Workshop on CLL (iwCLL) Guidelines (Hallek et al., 2018). PB samples were obtained following informed consent from patients enrolled in the CLL-BIO study, which was approved by the San Raffaele Hospital ethics committee. The clinical and biological characteristics of the enrolled patients are provided in Table S1. Any clinical investigation was conducted according to the principles expressed in the Declaration of Helsinki.

### Resources and reagents

Please see Table S2 for information on resources and reagents, including antibody dilutions and suppliers.

### Patient cell isolation and culture

Peripheral blood mononuclear cells isolated from patients with CLL (CLL-PBMCs) were separated via density gradient centrifugation (Lymphoprep). Purified CLL cells (CLL-PUR) were obtained by negative selection using a RosetteSep B lymphocyte enrichment kit. Cells were cultured at 37°C in 5% CO_2_ in AIM-V Medium (1×) supplemented with 10% v/v fetal bovine serum (FBS) and 5% v/v human serum (HS). CLL-PUR preparations consistently showed >95% purity, with co-expression of CD19 and CD5 confirmed by flow cytometry (NAVIOS, Beckman Coulter).

### 3D bone marrow microenvironment generation

The 3D BM microenvironment model was generated following a previously described protocol (Belloni et al., 2022). BM-derived stromal cells (HS-5) were seeded in the scaffolds (200×10^3^ cells per scaffold in 20 µl) and incubated for 4 h in static conditions at 37°C and 5% CO_2_ in an incubator. Scaffolds were then transferred to 10 ml high aspect ratio vessels (HARVs) in 600 μl pre-warmed Dulbecco's modified Eagle medium (DMEM) with 10% FBS and cultured overnight in an RCCS^TM^ bioreactor at the lowest speed (7.3 rpm). After the overnight dynamic seeding, PBMCs from CLL patients were added to the vessels (2×10^6^ cell/scaffold), and, after 5 h of dynamic seeding, vessels were filled with complete medium [AIM-V Medium (1×) with 10% FBS and 5% HS] and cultured in the bioreactor.

### 3D lymph node microenvironment generation

The 3D LN microenvironment was generated as previously described (Belloni et al., 2022), with the following adaptations: gelatine sponge scaffolds (Spongostan^TM^) were cut into cylinders (4 mm diameter×3.5 mm height). Human lymphatic fibroblasts (HLFs) and human umbilical vein endothelial cells (HUVECs) were seeded onto scaffolds at a 1:1 or 1:2 ratio (depending on HUVEC proliferation rate), using 2×10^5^ total cells in 20 µl per scaffold. Scaffolds were incubated at 37°C under static conditions for 3 h. CLL-PBMCs (2×10^6^ per scaffold in 500 µl) were then added to HARVs containing the populated scaffolds for overnight dynamic seeding at the lowest speed (7.3 rpm). The following day, vessels were filled with 11 ml pre-warmed complete medium (50% AIM-V and 50% EGM2, supplemented with 10% FBS and 5% HS) and maintained in the bioreactor under standard incubation conditions for 5 days. At the end of the culture period, cells were retrieved by enzymatic digestion using Liberase TL (25 µg/ml) and hyaluronidase (300 µg/ml), followed by flow cytometry analysis. Alternatively, scaffolds were fixed overnight in 4% paraformaldehyde (PFA), for imaging analysis. In parallel, adopting the same culture conditions as the 3D cell cultures, 2D co-culture conditions were established in multi-well plates using stromal cells and PBMCs at a 1:10 ratio (typically 2×10^5^ stromal cells and 2×10^6^ PBMCs per well across six wells).

### CLL cell proliferation assessment

CLL-PBMCs or CLL-PUR were stained with either Vybrant^TM^ CFDA-SE (0.5 µM) or CellTrace Violet^TM^ (5 µM) prior to stimulation with proliferative stimuli including CpG-ODN2006 (1 μg/ml) and IL-2 (50 ng/ml). Staining protocols followed manufacturer instructions and were optimized for cell densities up to 1×10^6^ cells/ml. To assess proliferation, cells were cultured for 5 days under one of the following conditions: 2D mono-culture (CLL-PUR or CLL-PBMCs) at 4×10^6^ cells/ml in 24- or 48-well plates; 2D co-culture with LN stroma (2×10^5^ stromal cells and 2×10^6^ PBMCs in 3 ml in six-well plates); or 3D co-culture in the bioreactor with LN scaffolds. Following culture, cells were harvested and analysed by flow cytometry for proliferation of CD19^+^CD5^+^ cells.

### Assessment of CLL cell mobilization

Ibrutinib (ibr; 1 µM) was administered to 3D CLL-PBMC co-cultures (LN and BM), following an adaptation of a previously described protocol (Belloni et al., 2022). After 5 days, cells were harvested and analysed by flow cytometry to assess mobilization.

### CLL cell viability assay

Venetoclax (ven; 2.5, 10, 20, 50 nM) was administered after 24 h from CLL cell seeding to 2D cultures of CLL-PBMCs, or to 2D and 3D co-cultures of CLL-PBMCs with LN or BM stroma. After 48 h of treatment, cell viability was assessed by flow cytometry using either Fixable Viability Dye eFluor^TM^ 660 or Annexin V/PI staining.

### Flow cytometric analysis

PBMCs from patients with CLL were characterized by flow cytometry using antibodies against CD19-ECD, CD5-PC7 and CD3-FITC, CXCR4-PE. In addition, to evaluate cell viability, staining with the Annexin-V/PI kit or with Fixable Viability Dye eFluor^TM^ 660 was performed. All samples were analysed using the multi-colour flow cytometer (NAVIOS, Beckman and Coulter) and successively analysed with FCS Express software (De Novo Software).

### Scaffold processing for image analysis

At the end of the experiment, scaffolds were fixed overnight in 4% PFA then incubated with 30% sucrose (in PBS) for 45 min. The scaffold was then included in OCT (BioOptica), fast frozen in isopentane and dry ice, and then stored at −80°C. The included samples were cut with a cryostat in slices of 7 μm thickness for histochemistry and 30 μm thickness for confocal reconstructions.

### Histochemistry

For the Haematoxylin Eosin staining, the sections were rehydrated with distilled water, stained with Haematoxylin, washed under running water and soaked in 95% ethanol, stained with Eosin, washed, and then dehydrated with 95% ethanol, 100% ethanol and xylene.

### Immunofluorescence

Slices cut from the scaffolds were permeabilized in blocking solution (0.1% w/v bovine serum albumin, 10% v/v FBS in PBS), containing 0.3% v/v Triton^TM^ X-100 for 30 min at room temperature (RT), and afterwards washed with PBS. Primary antibodies were diluted in blocking solution and incubated on the slices overnight at 4°C. Secondary antibodies were diluted in blocking solution and incubated on the slices for 2 h at RT; nuclei were stained with Hoechst 33342 for 10 min at RT. For the immunofluorescent staining, the following antibodies were used: anti-CD3, anti-CD31, anti-CD45, anti-CD68, anti-podoplanin and anti-ClCas3. For the secondary antibodies, we used Alexa Fluor 488 or Alexa Fluor 647. Actin was stained using Alexa Fluor 568 Phalloidin, added together with the secondary antibodies. For the detection of the ECM, the following reagents were used: Alexa Fluor^TM^ 488-conjugated Collagen IV, Alexa Fluor^TM^ 488-conjugated fibronectin monoclonal antibody or hyaluronic acid binding protein biotinylated. As secondary antibody for the detection of hyaluronic acid (HA), we used Alexa Fluor™ 647-conjugated Streptavidin. To exclude that the analysed ECMs were already present inside the scaffold, we set up a control by also staining the empty scaffold (Fig. S1). All images were acquired using an Olympus FluoVIEW 3000 RS confocal laser scanning microscope, equipped with the acquisition software FluoView Software FV31S. All images were acquired with a resolution of 1024×1024 pixels and analysed using ImageJ software. The objectives used were as follows: 10× (0.40 NA, dry), 20× (0.8 NA, dry), 30× (1.05 NA, silicone) and 60× (1.3 NA, silicone).

### Quantitative image analysis

The quantification of ECM proteins deposition was performed on 2D or 3D reconstructed images acquired at confocal microscopy (as previously described) using Fiji ImageJ software. The images containing the specific signal were adjusted as follows: rolling ball radius, 30 pixels; gaussian blur, 0.7 (radius). A default threshold algorithm was applied, and the area occupied by the signal was measured including only thresholded pixels. For the 3D reconstructed images, this process was applied to all the planes of the stack, and the volume (*V*) was computed as the sum of the occupied area *Ak* in each imaged slice *k*, multiplied by the *z*-step Δ*z*:

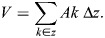


To make the comparison among 2D and 3D images possible, we normalized both data (2D measured occupied surface and 3D measured occupied volume) on the imaged surface area.

### Assessment of CLL cell mobilization

For the assessment of cell mobilization associated with ibr, an adaptation of the protocol from Barbaglio et al. (2021) was utilized. Mobilization induced by ibr was assessed in both the BM and LN models. After 48 h or 72 h of 3D dynamic culture in the bioreactor, supernatants were collected from the vessels, centrifuged at 410 ***g*** for 5 min, and the recovered cells were counted and used as control. The clarified supernatants were put back into the vessels, with or without 1 μM ibr. Cell mobilization was then assessed after 5 h by counting the cells recovered from the supernatant.

### Statistical analyses

Statistical analyses were performed using GraphPad Prism software. Data in Fig. 1 are presented as mean±s.e.m. In all other figures, data are presented as Tukey box-and-whisker plots. The middle line represents the median, the box represents the interquartile range (25th–75th percentiles), and the whiskers represent 1.5 times the interquartile range. Individual points outside the whiskers represent biological outliers. Key paired comparisons are additionally visualized as dot plots with lines connecting measurements from the same subject. For paired data, normality was assessed on the distribution of paired differences using Shapiro–Wilk test and visual inspection of Q–Q plots. Statistical tests used depended on the distribution of the different datasets and are specified in figure legends. *P*<0.05 was considered significant.

## Supplementary Material

10.1242/dmm.052731_sup1 Supplementary information

## References

[DMM052731C1] Anderson, N. M. and Simon, M. C. (2020). The tumor microenvironment. *Curr. Biol.* 30, R921-R925. 10.1016/j.cub.2020.06.081 32810447 PMC8194051

[DMM052731C2] Anderson, M. A., Deng, J., Seymour, J. F., Tam, C., Kim, S. Y., Fein, J., Yu, L., Brown, J. R., Westerman, D., Si, E. G. et al. (2016). The BCL2 selective inhibitor venetoclax induces rapid onset apoptosis of CLL cells in patients via a TP53-independent mechanism. *Blood* 127, 3215-3224. 10.1182/blood-2016-01-688796 27069256 PMC4920022

[DMM052731C3] Anderson, M. A., Walewska, R., Hackett, F., Kater, A. P., Montegaard, J., O'Brien, S., Seymour, J. F., Smith, M., Stilgenbauer, S., Whitechurch, A. et al. (2024). Venetoclax initiation in chronic lymphocytic leukemia: international insights and innovative approaches for optimal patient care. *Cancers* 16, 980. 10.3390/cancers16050980 38473342 PMC10931175

[DMM052731C4] Bahramsoltani, M., Slosarek, I., De Spiegelaere, W. and Plendl, J. (2014). Angiogenesis and collagen type IV expression in different endothelial cell culture systems. *Anat. Histol. Embryol.* 43, 103-115. 10.1111/ahe.12052 23551189

[DMM052731C5] Barbaglio, F., Belloni, D., Scarfò, L., Sbrana, F. V., Ponzoni, M., Bongiovanni, L., Pavesi, L., Zambroni, D., Stamatopoulos, K., Caiolfa, V. R. et al. (2021). Three-dimensional co-culture model of chronic lymphocytic leukemia bone marrow microenvironment predicts patient-specific response to mobilizing agents. *Haematologica* 106, 2334-2344. 10.3324/haematol.2020.248112 32732361 PMC8409046

[DMM052731C6] Barozzi, D. and Scielzo, C. (2023). Emerging strategies in 3D culture models for hematological cancers. *Hemasphere* 7, E932. 10.1097/HS9.0000000000000932 37520775 PMC10378728

[DMM052731C7] Barozzi, D., Scagnoli, F., Barbaglio, F., Belloni, D., Ribezzi, D., Farè, S., Berno, V., Pinos, R., Sampietro, M., Pauri, M. et al. (2025). Dynamic stimulation promotes functional tissue-like organization of a 3D human lymphoid microenvironment model in vitro. *Cell Rep. Methods* 5, 101105. 10.1016/j.crmeth.2025.101105 40651477 PMC12296514

[DMM052731C8] Belloni, D., Heltai, S., Ponzoni, M., Villa, A., Vergani, B., Pecciarini, L., Marcatti, M., Girlanda, S., Tonon, G., Ciceri, F. et al. (2018). Modeling multiple myeloma-bone marrow interactions and response to drugs in a 3d surrogate microenvironment. *Haematologica* 103, 707-716. 10.3324/haematol.2017.167486 29326121 PMC5865414

[DMM052731C9] Belloni, D., Ferrarini, M., Ferrero, E., Guzzeloni, V., Barbaglio, F., Ghia, P. and Scielzo, C. (2022). Protocol for generation of 3D bone marrow surrogate microenvironments in a rotary cell culture system. *STAR Protoc.* 3, 101601. 10.1016/j.xpro.2022.101601 35990738 PMC9382330

[DMM052731C10] Bertilaccio, M. T. S. and Chen, S. S. (2024). Mouse models of chronic lymphocytic leukemia and Richter transformation: what we have learnt and what we are missing. *Front. Immunol.* 15, 1376660. 10.3389/fimmu.2024.1376660 38903501 PMC11186982

[DMM052731C11] Brachtl, G., Piñón Hofbauer, J., Greil, R. and Hartmann, T. N. (2014). The pathogenic relevance of the prognostic markers CD38 and CD49d in chronic lymphocytic leukemia. *Ann. Hematol*. 93, 361-374. 10.1007/s00277-013-1967-y 24288111 PMC4032465

[DMM052731C12] Burger, J. A. (2011). Nurture versus nature: the microenvironment in chronic lymphocytic leukemia. *Hematology Am. Soc. Hematol. Educ. Program.* 2011, 96-103. 10.1182/asheducation-2011.1.96 22160019

[DMM052731C13] Burger, J. A. and O'Brien, S. (2018). Evolution of CLL treatment — from chemoimmunotherapy to targeted and individualized therapy. *Nat. Rev. Clin. Oncol*. 15, 510-527. 10.1038/s41571-018-0037-8 29777163

[DMM052731C14] Burger, J. A., Tsukada, N., Burger, M., Zvaifler, N. J., Dell'aquila, M. and Kipps, T. J. (2000). Blood-derived nurse-like cells protect chronic lymphocytic leukemia B cells from spontaneous apoptosis through stromal cell-derived factor-1. *Blood* 96, 2655-2663. 10.1182/blood.V96.8.2655 11023495

[DMM052731C15] Burger, J. A., Ghia, P., Rosenwald, A. and Caligaris-Cappio, F. (2009). The microenvironment in mature B-cell malignancies: a target for new treatment strategies. *Blood* 114, 3367-3375. 10.1182/blood-2009-06-225326 19636060 PMC4969052

[DMM052731C16] Caligaris-Cappio, F. (2014). Anergy: the CLL cell limbo. *Adv. Exp. Med. Biol.* 123, 3214-3215. 10.1182/blood-2014-04-565408 24855189

[DMM052731C17] Caligaris-Cappio, F. and Hamblin, T. J. (1999). B-cell chronic lymphocytic leukemia: a bird of a different feather. *J. Clin. Oncol.* 17, 399-408. 10.1200/JCO.1999.17.1.399 10458259

[DMM052731C18] Caligaris-Cappio, F., Bertilaccio, M. T. S. and Scielzo, C. (2014). How the microenvironment wires the natural history of chronic lymphocytic leukemia. *Semin. Cancer Biol.* 24, 43-48. 10.1016/j.semcancer.2013.06.010 23831274

[DMM052731C19] Calissano, C., Damle, R. N., Marsilio, S., Yan, X. J., Yancopoulos, S., Hayes, G., Emson, C., Murphy, E. J., Hellerstein, M. K., Sison, C. et al. (2011). Intraclonal complexity in chronic lymphocytic leukemia: fractions enriched in recently born/divided and older/quiescent cells. *Mol. Med.* 17, 1374-1382. 10.2119/molmed.2011.00360 21968788 PMC3321822

[DMM052731C20] Chen, S. and Chiorazzi, N. (2023). Functional consequences of inhibition of Bruton's tyrosine kinase by ibrutinib in chronic lymphocytic leukemia. *Hematol. Oncol.* 41, 119-128. 10.1002/hon.3144 37294973

[DMM052731C21] Dal Bo, M., Tissino, E., Benedetti, D., Caldana, C., Bomben, R., Del Poeta, G., Gaidano, G., Rossi, F. M., Zucchetto, A. and Gattei, V. (2014). Microenvironmental interactions in chronic lymphocytic leukemia: the master role of CD49d. *Semin. Hematol.* 51, 168-176. 10.1053/j.seminhematol.2014.05.002 25048781

[DMM052731C22] D'Arena, G., Calapai, G. and Deaglio, S. (2014). Anti-CD44 mAb for the treatment of B-cell chronic lymphocytic leukemia and other hematological malignancies: evaluation of WO2013063498. *Expert. Opin. Ther. Pat.* 24, 821-828. 10.1517/13543776.2014.915942 24798704

[DMM052731C23] De, M.-T., Fuente, L., Casanova, B., Garcia-Gila, M., Silva, A. and Garcia-Pardo, A. (1999). Fibronectin interaction with alpha4beta1 integrin prevents apoptosis in B cell chronic lymphocytic leukemia: correlation with Bcl-2 and Bax, Leukemia. *Leukemia* 13, 266-274. 10.1038/sj.leu.2401275 10025901

[DMM052731C24] Deaglio, S., Aydin, S., Grand, M. M., Vaisitti, T., Bergui, L., D'Arena, G., Chiorino, G. and Malavasi, F. (2010). CD38/CD31 interactions activate genetic pathways leading to proliferation and migration in chronic lymphocytic leukemia cells. *Mol. Med.* 16, 87-91. 10.2119/molmed.2009.00146 19956559 PMC2785473

[DMM052731C25] Dubois, N., Crompot, E., Meuleman, N., Bron, D., Lagneaux, L. and Stamatopoulos, B. (2020). Importance of crosstalk between chronic lymphocytic leukemia cells and the stromal microenvironment: direct contact, soluble factors, and extracellular vesicles. *Front. Oncol.* 10, 1422. 10.3389/fonc.2020.01422 32974152 PMC7466743

[DMM052731C26] Eichhorst, B., Niemann, C. U., Kater, A. P., Fürstenau, M., von Tresckow, J., Zhang, C., Robrecht, S., Gregor, M., Juliusson, G., Thornton, P. et al. (2023). First-line venetoclax combinations in chronic lymphocytic leukemia. *N. Engl. J. Med.* 388, 1739-1754. 10.1056/nejmoa2213093 37163621

[DMM052731C27] Elias, E. E., Sarapura Martinez, V. J., Amondarain, M., Colado, A., Cordini, G., Bezares, R. F., Fernandez Grecco, H., Custidiano, M. R., Sánchez Ávalos, J. C., Garate, G. et al. (2022). Venetoclax-resistant CLL cells show a highly activated and proliferative phenotype. *Cancer Immunol. Immunother.* 71, 979-987. 10.1007/s00262-021-03043-x 34467417 PMC10992976

[DMM052731C28] Friedman, D., Mehtani, D. P., Vidler, J. B., Patten, P. E. M. and Hoogeboom, R. (2024). Proliferating CLL cells express high levels of CXCR4 and CD5. *Hemasphere* 8, e70064. 10.1002/hem3.70064 39691453 PMC11651208

[DMM052731C29] Galletti, G., Caligaris-Cappio, F. and Bertilaccio, M. T. S. (2016). B cells and macrophages pursue a common path toward the development and progression of chronic lymphocytic leukemia. *Leukemia*. 30, 2293-2301. 10.1038/leu.2016.261 27677742

[DMM052731C30] Girbl, T., Hinterseer, E., Grössinger, E. M., Asslaber, D., Oberascher, K., Weiss, L., Hauser-Kronberger, C., Neureiter, D., Kerschbaum, H., Naor, D. et al. (2013). CD40-mediated activation of chronic lymphocytic leukemia cells promotes their CD44-dependent adhesion to hyaluronan and restricts CCL21-induced motility. *Cancer Res.* 73, 561-570. 10.1158/0008-5472.CAN-12-2749 23117883

[DMM052731C31] Hallek, M. (2025). Chronic lymphocytic leukemia: 2025 update on the epidemiology, pathogenesis, diagnosis, and therapy. *Am. J. Hematol.* 100, 450-480. 10.1002/ajh.27546 39871707 PMC11803567

[DMM052731C32] Hallek, M., Cheson, B. D., Catovsky, D., Caligaris-Cappio, F., Dighiero, G., Hillmen, P., Keating, M., Montserrat, E., Chiorazzi, N., Stilgenbauer, S. et al. (2018). Special report iwCLL guidelines for diagnosis, indications for treatment, response assessment, and supportive management of CLL. *Blood* 131, 2745-2760. 10.1182/blood-2017-09-806398 29540348

[DMM052731C33] Haselager, M. V., Kielbassa, K., Ter Burg, J., Bax, D. J. C., Fernandes, S. M., Borst, J., Tam, C., Forconi, F., Chiodin, G., Brown, J. R. et al. (2020). Changes in Bcl-2 members after ibrutinib or venetoclax uncover functional hierarchy in determining resistance to venetoclax in CLL. *Blood* 136, 2918-2926. 10.1182/blood.2019004326 32603412

[DMM052731C34] Haselager, M. V., Van Driel, B. F., Perelaer, E., De Rooij, D., Lashgari, D., Loos, R., Kater, A. P., Moerland, P. D. and Eldering, E. (2023). In vitro 3D spheroid culture system displays sustained T cell-dependent CLL proliferation and survival. *Hemasphere* 7, E938. 10.1097/HS9.0000000000000938 37637994 PMC10448932

[DMM052731C35] Herishanu, Y., Pé Rez-Galán, P., Liu, D., Lique Biancotto, A., Pittaluga, S., Vire, B., Gibellini, F., Njuguna, N., Lee, E., Stennett, L. et al. (2011a). The lymph node microenvironment promotes B-cell receptor signaling, NF-B activation, and tumor proliferation in chronic lymphocytic leukemia. *Blood* 117, 563-574. 10.1182/blood-2010-05 20940416 PMC3031480

[DMM052731C64] Herishanu, Y., Gibellini F., Njuguna, N., Hazan-Halevy, I., Farooqui, M., Bern, S., Keyvanfar, K., Lee, E., Wilson, W. and Wiestner, A. (2011b). Activation of CD44, a receptor for extracellular matrix components, protects chronic lymphocytic leukemia cells from spontaneous and drug induced apoptosis through MCL-1. *Leuk. Lymphoma* 52, 1758-1769. 10.3109/10428194.2011.569962 21649540 PMC3403533

[DMM052731C36] Herishanu, Y., Katz, B. Z., Lipsky, A. and Wiestner, A. (2013). Biology of chronic lymphocytic leukemia in different microenvironments. Clinical and therapeutic implications. *Hematol. Oncol. Clin. North Am.* 27, 173-206. 10.1016/j.hoc.2013.01.002 23561469 PMC3660068

[DMM052731C37] Herman, S. E. M., Niemann, C. U., Farooqui, M., Jones, J., Mustafa, R. Z., Lipsky, A., Saba, N., Martyr, S., Soto, S., Valdez, J. et al. (2014). Ibrutinib-induced lymphocytosis in patients with chronic lymphocytic leukemia: correlative analyses from a phase II study. *Leukemia* 28, 2188-2196. 10.1038/leu.2014.122 24699307 PMC4185271

[DMM052731C38] Herndon, T. M., Chen, S. S., Saba, N. S., Valdez, J., Emson, C., Gatmaitan, M., Tian, X., Hughes, T. E., Sun, C., Arthur, D. C. et al. (2017). Direct in vivo evidence for increased proliferation of CLL cells in lymph nodes compared to bone marrow and peripheral blood. *Leukemia* 31, 1340-1347. 10.1038/leu.2017.11 28074063 PMC5462849

[DMM052731C39] Kater, A. P., Owen, C., Moreno, C., Follows, G., Munir, T., Levin, M.-D., Benjamini, O., Janssens, A., Osterborg, A., Robak, T. et al. (2022). Fixed-duration ibrutinib-venetoclax in patients with chronic lymphocytic leukemia and comorbidities. *NEJM Evidence* 1. EVIDoa2200006. 10.1056/evidoa2200006 38319255

[DMM052731C40] Kim, H. N., Ogana, H., Sanchez, V., Nichols, C. and Kim, Y. M. (2022). PI3K targeting in non-solid cancer. *Curr. Top. Microbiol. Immunol.* 436, 393-407. 10.1007/978-3-031-06566-8_17 36243854 PMC10075235

[DMM052731C41] Kipps, T. J., Stevenson, F. K., Wu, C. J., Croce, C. M., Packham, G., Wierda, W. G., O'Brien, S., Gribben, J. and Rai, K. (2017). Chronic lymphocytic leukaemia. *Nat. Rev. Dis. Primers* 3, 16096. 10.1038/nrdp.2016.96 28102226 PMC5336551

[DMM052731C42] Kramer, R. H., Rosen, S. D. and Mcdonald, K. A. (1988). Basement-membrane components associated with the extracellular matrix of the lymph node. *Cell Tissue Res.* 252, 367-375. 10.1007/BF00214379 3383216

[DMM052731C43] Lenti, E., Visentin, E., Bojnik, E., Neroni, A., Franchino, M., Talarico, D., Sacchetti, N., Scarfò, L., Maurizio, A., Garcia-Manteiga, J. M. et al. (2025). A spheroid model that recapitulates the protective role of the lymph node microenvironment and serves as a platform for drug testing in chronic lymphocytic leukemia. *Hemasphere* 9, e70170. 10.1002/hem3.70170 40625531 PMC12233836

[DMM052731C44] Mazzarello, A. N., Fitch, M., Cardillo, M., Ng, A., Bhuiya, S., Sharma, E., Bagnara, D., Kolitz, J. E., Barrientos, J. C., Allen, S. L. et al. (2023). Characterization of the intraclonal complexity of chronic lymphocytic leukemia B cells: potential influences of B-cell receptor crosstalk with other stimuli. *Cancers* 15, 4706. 10.3390/cancers15194706 37835400 PMC10571896

[DMM052731C45] Minici, C., Gounari, M., Übelhart, R., Scarfò, L., Dühren-von Minden, M., Schneider, D., Tasdogan, A., Alkhatib, A., Agathangelidis, A., Ntoufa, S. et al. (2017). Distinct homotypic B-cell receptor interactions shape the outcome of chronic lymphocytic leukaemia. *Nat. Commun.* 8, 15746. 10.1038/ncomms15746 28598442 PMC5472768

[DMM052731C46] Newman, P. J. (1997). Perspectives series: cell adhesion in vascular biology the biology of PECAM-1. *J. Clin. Invest* 99, 2814-2817. 10.1172/JCI119129 9185501 PMC508129

[DMM052731C47] Ntoufa, S., Vilia, M. G., Stamatopoulos, K., Ghia, P. and Muzio, M. (2016). Toll-like receptors signaling: a complex network for NF-κB activation in B-cell lymphoid malignancies. *Semin. Cancer Biol.* 39, 15-25. 10.1016/j.semcancer.2016.07.001 27402288

[DMM052731C48] Pasikowska, M., Walsby, E., Apollonio, B., Cuthill, K., Phillips, E., Coulter, E., Longhi, M. S., Ma, Y., Yallop, D., Barber, L. D. et al. (2016). Phenotype and immune function of lymph node and peripheral blood CLL cells are linked to transendothelial migration. *Blood* 128, 563-573. 10.1182/blood-2016-01-683128 27252234

[DMM052731C49] Patten, J. and Wang, K. (2021). Fibronectin in development and wound healing. *Adv. Drug Deliv. Rev.* 170, 353-368. 10.1016/j.addr.2020.09.005 32961203

[DMM052731C50] Ponzoni, M., Doglioni, C. and Caligaris-Cappio, F. (2011). Chronic lymphocytic leukemia: the pathologist's view of lymph node microenvironment. *Semin. Diagn. Pathol.* 28, 161-166. 10.1053/j.semdp.2011.02.014 21842701

[DMM052731C51] Primo, D., Scarfò, L., Xochelli, A., Mattsson, M., Ranghetti, P., Belén Espinosa, A., Robles, A., Gorrochategui, J., Martínez-López, J., De La Serna, J. et al. (2018). A novel ex vivo high-throughput assay reveals antiproliferative effects of idelalisib and ibrutinib in chronic lymphocytic leukemia. *Oncotarget* 9, 26019-26031. 10.18632/oncotarget.25419 29899839 PMC5995261

[DMM052731C52] Roessner, P. M. and Seiffert, M. (2020). T-cells in chronic lymphocytic leukemia: guardians or drivers of disease? *Leukemia* 34, 2012-2024. 10.1038/s41375-020-0873-2 32457353 PMC8318881

[DMM052731C53] Roet, J. E. G., Morrison, A. I., Mikula, A. M., de Kok, M., Panocha, D., Roest, H. P., van der Laan, L. J. W., de Winde, C. M. and Mebius, R. E. (2024). Human lymph node fibroblastic reticular cells maintain heterogeneous characteristics in culture. *iScience* 27, 110179. 10.1016/j.isci.2024.110179 38989462 PMC11233964

[DMM052731C54] Sampietro, M., Cellani, M. and Scielzo, C. (2025). B cell mechanobiology in health and disease: emerging techniques and insights into therapeutic responses. *FEBS Lett.* 599, 2854-2877. 10.1002/1873-3468.70071 40387441 PMC12558679

[DMM052731C55] Scarfò, L., Ferreri, A. J. M. and Ghia, P. (2016). Chronic lymphocytic leukaemia. *Crit. Rev. Oncol. Hematol*. 104, 169-182. 10.1016/j.critrevonc.2016.06.003 27370174

[DMM052731C56] Scielzo, C. and Ghia, P. (2020). Modeling the leukemia microenviroment in vitro. *Front. Oncol.* 10. 607608. 10.3389/fonc.2020.607608 33392097 PMC7773937

[DMM052731C57] Skanland, S. S. and Mato, A. R. (2021). Overcoming resistance to targeted therapies in chronic lymphocytic leukemia. *Blood Adv*. 5, 334-343. 10.1182/BLOODADVANCES.2020003423 33570649 PMC7805313

[DMM052731C58] Takács, F., Mikala, G., Nagy, N., Reszegi, A., Czeti, Á., Szalóki, G. and Barna, G. (2021). Identification of a novel resistance mechanism in venetoclax treatment and its prediction in chronic lymphocytic leukemia. *Acta Oncol.* 60, 528-530. 10.1080/0284186X.2021.1878388 33491510

[DMM052731C59] Ten Hacken, E. and Burger, J. A. (2014). Microenvironment dependency in Chronic Lymphocytic Leukemia: the basis for new targeted therapies. *Pharmacol. Ther*. 144, 338-348. 10.1016/j.pharmthera.2014.07.003 25050922

[DMM052731C60] ten Hacken, E., Gounari, M., Ghia, P. and Burger, J. A. (2019). The importance of B cell receptor isotypes and stereotypes in chronic lymphocytic leukemia. *Leukemia* 33, 287-298. 10.1038/s41375-018-0303-x 30555163 PMC7182338

[DMM052731C61] Yu, X., Munoz-Sagredo, L., Streule, K., Muschong, P., Bayer, E., Walter, J.,R., Gutjahr, C.,J., Greil, R., Concha, L.,M., Muller-Tidow, C. et al. (2020). CD44 loss of function sensitizes AML cells to the BCL-2 inhibitor venetoclax by decreasing CXCL12-driven survival cues. *Blood* 136, 2125-2132. 10.1182/blood.2020006343 34115113

[DMM052731C62] Zhou, Z., Pausch, F., Schlötzer-Schrehardt, U., Brachvogel, B. and Pöschl, E. (2016). Induction of initial steps of angiogenic differentiation and maturation of endothelial cells by pericytes in vitro and the role of collagen IV. *Histochem. Cell Biol.* 145, 511-525. 10.1007/s00418-015-1398-z 26747274

[DMM052731C63] Zielonka, K. and Jamroziak, K. (2024). Mechanisms of resistance to venetoclax in hematologic malignancies. *Adv. Clin. Exp. Med*. 33, 1421-1433. 10.17219/acem/181145 38439610

