# Supplementary material for: A 3D lymph node model for chronic lymphocytic leukaemia recapitulates microenvironmental features and drug response in vitro
Source: Dis Model Mech. 2026 Mar 31;19(6):dmm052731. doi: 10.1242/dmm.052731 (PMC13072081; doi:10.1242/dmm.052731)
Supplement: Supplementary information [file dmm-19-052731-s1.pdf]

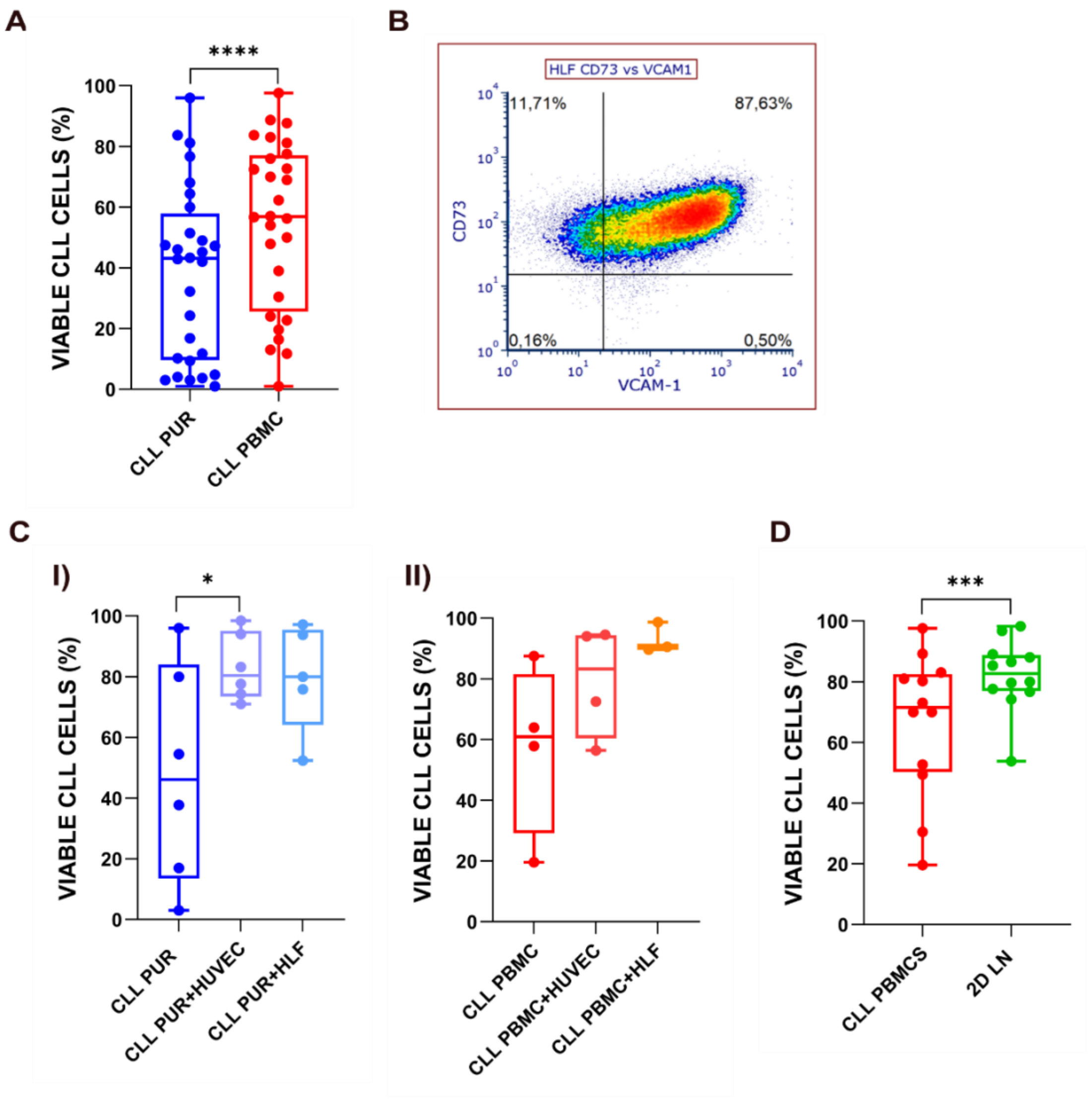

Fig. S1. Viability of CLL primary cells

Evaluation of CLL cell viability by flow cytometry. **(A)** Viability of CLL-PUR and CLL-PBMCs mono-cultures after 5 days ($n = 28$, Wilcoxon signed-rank test for paired samples, $p < 0.0001$). **(B)** Expression levels of CD73 and VCAM-1 in HLF cells. **(C)** Viability of **I-** CLL-PUR, CLL-PUR + HUVEC, CLL-PUR + HLF cultured in parallel or **II-** CLL-PBMCs, CLL-PBMCs + HUVEC, CLL-PBMCs + HLF (Mixed-effect analysis with Geisser-Greenohouse correction and Tukey's multiple comparisons test. I: $n = 6$, CLL PUR vs CLL PUR+HUVEC $p = 0.0488$; II: $n=4$) (**D**) Viability of CLL-PBMCs mono-cultures or co-cultures with the LN stroma ($n=12$, Wilcoxon signed-rank test for paired samples, $p = 0.0010$).

Disease Models & Mechanisms • Supplementary information

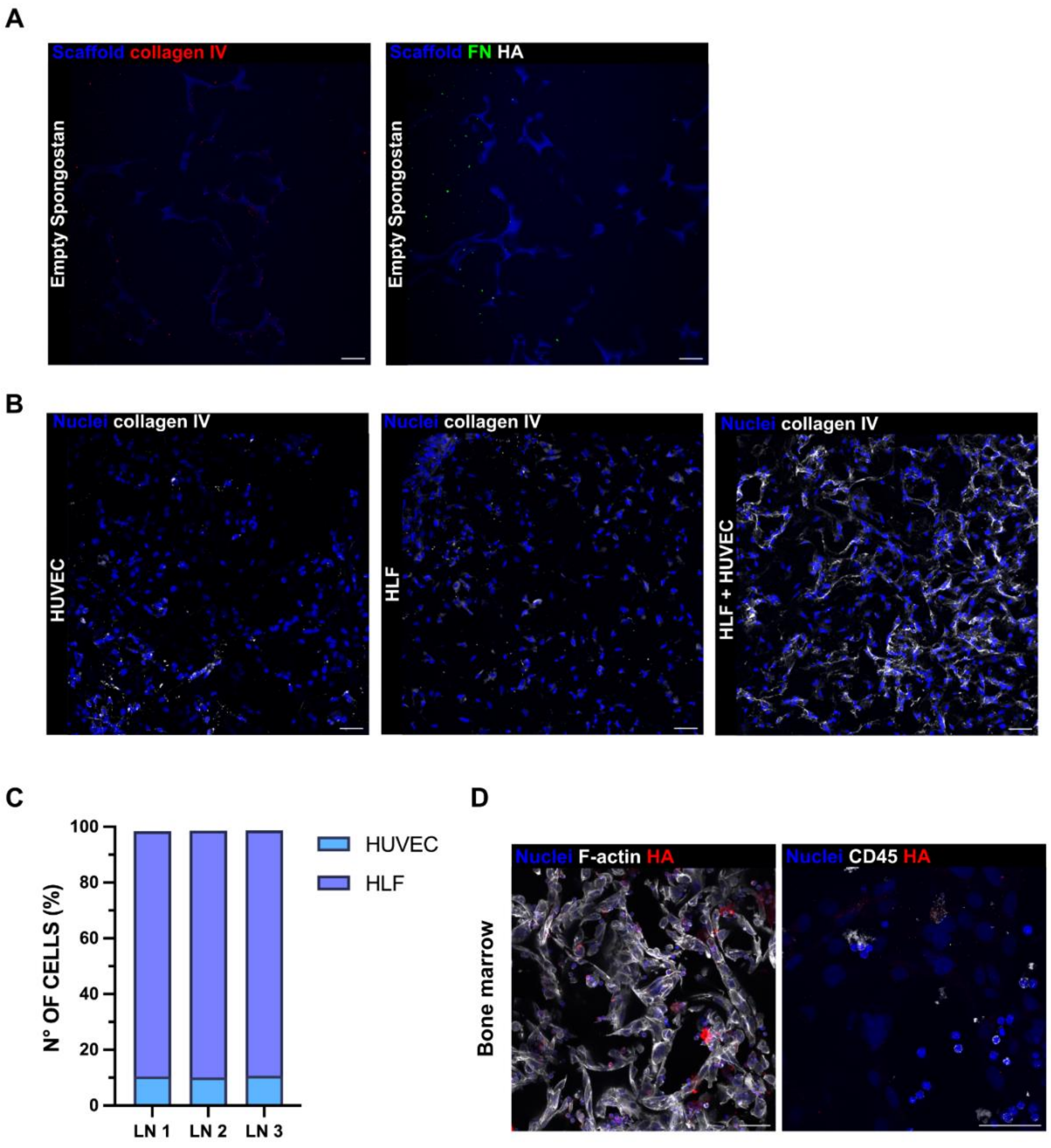

Fig. S2. Spongostan empty immunofluorescence control

(A) Immunofluorescence images of empty Spongostan after staining with Ab anti-ECM proteins as negative control. Spongostan is not an optical transparent material, in these images its autofluorescence was enhanced using the 405nm laserline to show the presence of the matrix in absence of ECM proteins (stained with Alexa 488, and Alexa 647 fluorophore-conjugated antibodies. See Supplementary Methods section). Scale bar 50 µm. **(B)** Immunofluorescence images of scaffolds populated with HUVEC alone **(left)**, HLF alone **(middle)** or both **(right)** after staining with collagen IV. Scale bar 50 µm. **(C)** Flow cytometry analysis of the HLF/HUVEC ratio in the 3D scaffold after dissociation. Cell types were discriminated based on CD31 and CD90 surface markers expression: HUVEC = $CD31^{+}$ $CD90^{-}$; HLF = $CD31^{-}$ $CD90^{+}$. **(D)** Immunofluorescence 3D reconstructions of the BM model. Scale bar 50 µm.

Disease Models & Mechanisms • Supplementary information

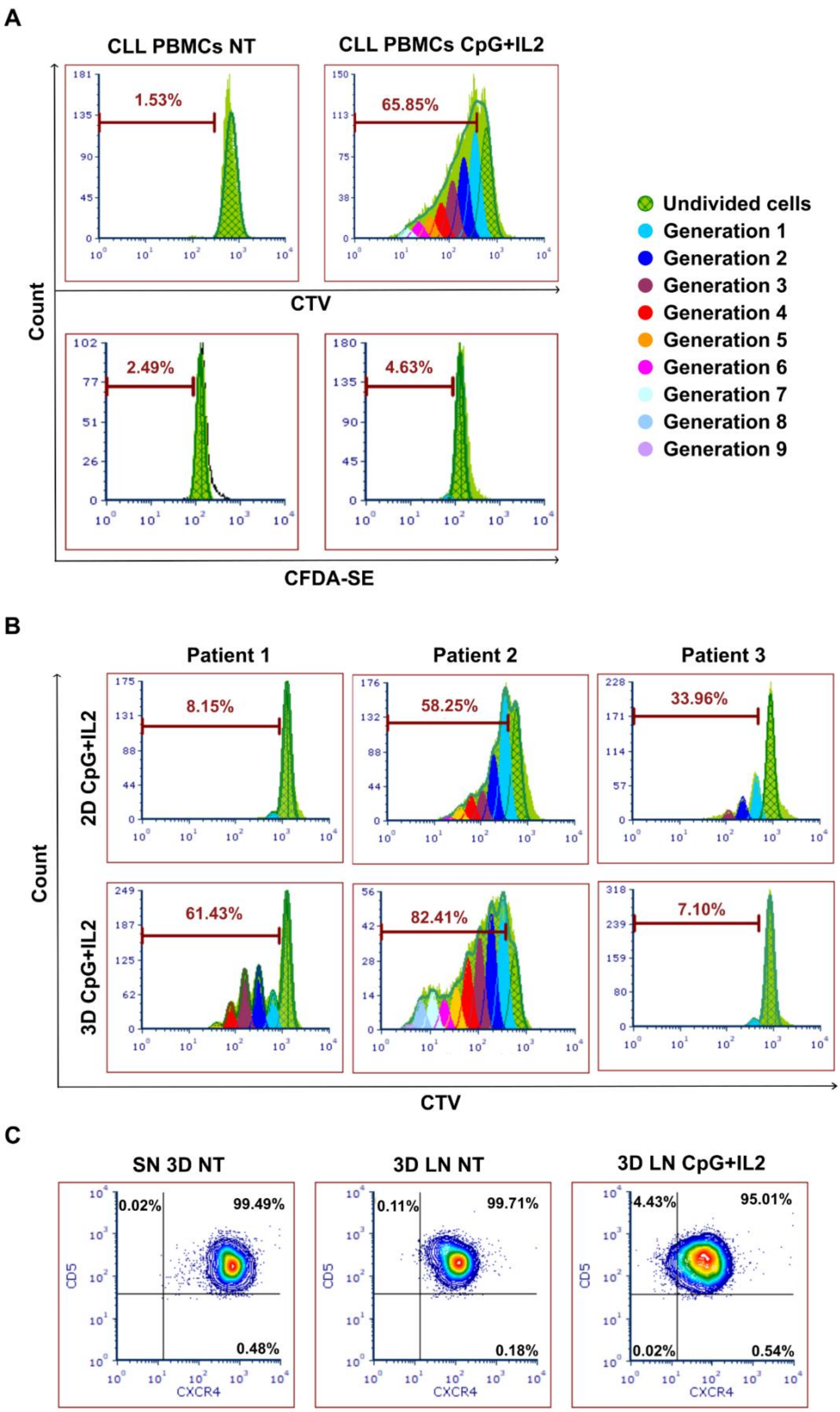

Disease Models & Mechanisms • Supplementary information

Fig. S3. Assessment of proliferation in presence or absence of LN stroma

(**A**) Example of the proliferation assessment by flow cytometry of 2D PBMCs mono-culture NT and in presence of proliferative stimuli. **Top**, proliferation assessed with the Cell Trace Violet (CTV); **Bottom**, proliferation assessed with CFDA-SE. **(B)** Example of the proliferation assessment by flow cytometry of 3 representative patients in 2D and 3D in presence of proliferative stimuli. The proliferation was evaluated with CTV. The legend in panel **(A)** refers to the peaks in both panels **(A)** and **(B)**, which discriminate multiple generational peaks corresponding to successive halving of dye fluorescence intensity, thereby indicating multiple rounds of cell division. **(C)** Flow cytometry density plots displaying CD5/CXCR4 expression of CLL PBMCs in 3D scaffold (SC) and surnatant (SN) NT, as well as in the SC in presence of proliferative stimuli.

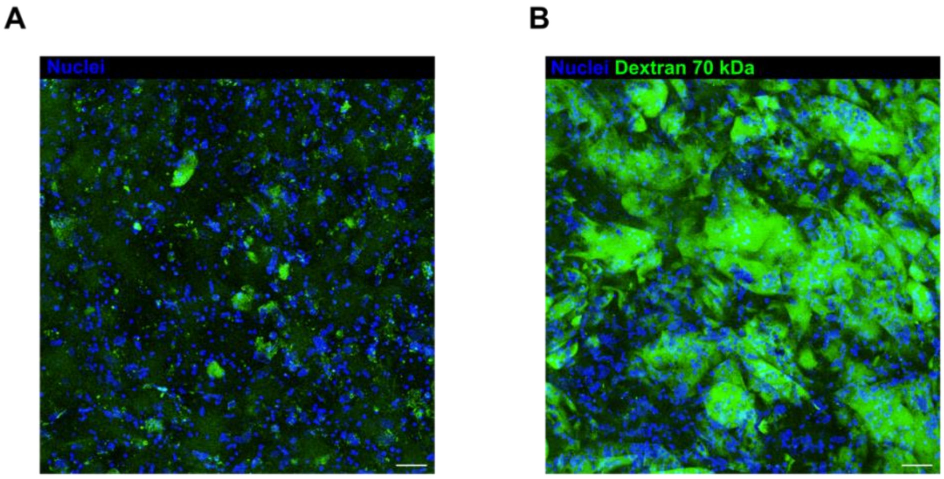

Fig. S4. Scaffold perfusion with Dextran-FITC

Confocal images of the scaffold permeability test with FITC-labelled Dextran after 5 days of dynamic culture and 3 hours of dynamic incubation with Dextran. The images were taken in the middle part of the scaffold. **(A)** Control in absence of FITC-labelled Dextran; the signal is relative to scaffold and cells autofluorescence **(B)** 70kDa FITC-labelled Dextran. Scale bar 50µm.

Disease Models & Mechanisms • Supplementary information

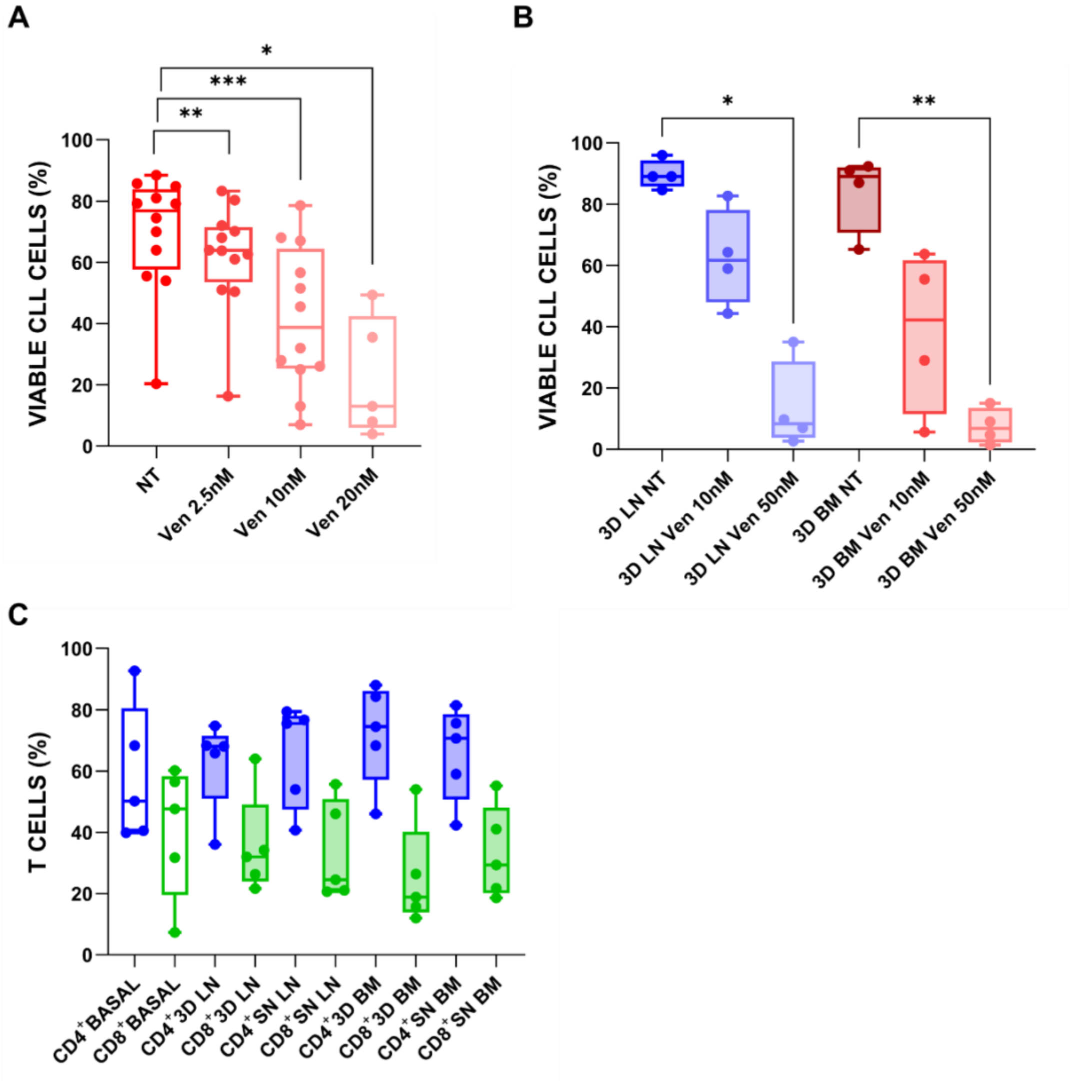

Fig. S5. Venetoclax titration

Viability evaluation by flow cytometry in different conditions. **(A)** Venetoclax titration on CLL-PBMCs (n = 12, Mixed-effect analysis with Geisser-Greenohouse correction and Dunnet's multiple comparisons test, NT vs Ven 2.5nM p = 0.0019; NT vs Ven 10nM p = 0.0008; NT vs Ven 20nM p = 0.0110). **(B)** CLL-PBMCs in the 3D LN and BM co-culture treatment NT and with venetoclax 10 nM and 50 nM (n=4, one-way ANOVA with Geisser-Greenhouse correction and Tukey's comparisons test, LN NT vs LN Ven 50nM p = 0.0103; BM NT vs BM Ven 50nM p = 0.0034). **(C) I**- Flow cytometry analysis highlighting the % of T cells from CLL PBMCs at basal conditions (from patient's blood), supernatant (SN) and 3D LN and BM scaffolds (n = 5); **II**- $CD4^{+}$ and $CD8^{+}$ populations in both SN and 3D LN and BM (n = 5); **III**- CD4/CD8 ratio at basal condition and in the SN and 3D LN and BM (n = 5).

Disease Models & Mechanisms • Supplementary information

Table S1. Patients' samples diagnostic and prognostic characteristics.

Patient	Gender	Stage at diagnosis	Progression state	CD38 (%)	IGHV	Germline identity (%)	Mutations	Subset	Light Chain
**PT1**	M	II	Progressive	5.9	IGHV4-31*03 F	96.22		NS	L
**PT2**	F	0	Stable	0	IGHV3-9*01 F	96.53		NS	K
**PT3**	M	NA	Progressive	2	IGHV4-34*01 F	96.14		NS	K
**PT4**	F	0	Stable	4.8	IGHV4-39*07 F	89.69		NS	K
**PT5**	M	0	Stable	37.5	IGHV3-21*01 F	97.57		#2	L
**PT6**	M	0	Stable	5	IGHV5-51*01 F	100		NS	L
**PT7**	M	0	Stable	0.1	Homsap IGHV5-51*01 F	100	del(11q)	NS	L
**PT8**	F	0	Stable	0	IGHV4-34*03 F, or IGHV4-34*06 F	90.49		#201	L
**PT9**	M	II	Stable	5	IGHV3-64*01 F	100	del(17p), del(13q)	NS	K
**PT10**	M	0	Stable	0.1	IGHV3-30*01 F	100		NS	L
**PT11**	F	0	Stable	NA	Homsap IGHV4-34*01 F	92.63		NS	L
**PT12**	M	NA	Progressive	93	IGHV4-34*01 F	96.49		NS	K
**PT13**	M	NA	Progressive	2	IGHV3-23*01 F or IGHV3-23*02 or IGHV3-23D*01	92.36		NS	L
**PT14**	M	NA	Stable	91.6	IGHV1-69*06 F	100		NS	L
**PT15**	M	NA	Stable	NA	Homsap IGHV4-4*02 F	91.23		#77	K
**PT16**	M	0	Progressive	5	IGHV5-51*01 F	100		NS	L
**PT17**	F	NA	Progressive	38.3	Homsap IGHV3-15*01 F, or Homsap IGHV3-15*07 F	97.28		NS	L
**PT18**	M	0	Progressive	71.5	Homsap IGHV1-69*01 F, or Homsap IGHV1-69D*0 F	100		NS	L
**PT19**	F	0	Stable	0	IGHV4-34*01F	95.09		#1	K
**PT20**	F	0	Stable	0	IGHV3-48*04F	94.1		NS	K
**PT21**	M	0	Progressive	1.2	IGHV3-7*01F	100	del(17p)	NS	L
**PT22**	M	0	Stable	NA	IGHV4-38-2*02 F	96.88	del(13q)	NS	K
**PT23**	F	0	Stable	0	Homsap IGHV4-4*10 (F)	88.54		#14	L
**PT24**	M	II	Stable	0	IGHV3-15*01 F	98.89		NS	L
**PT25**	F	0	Stable	0.1	IGHV3-7*01F	99.65		NS	L
**PT26**	F	0	Stable	0	Unproductive	Unproductive		NS	L
**PT27**	M	0	Stable	90.6	IGHV1-69*01 F, or	100		NS	K

Disease Models & Mechanisms • Supplementary information

					IGHV1-69D*01 F				
**PT28**	F	0	Stable	0	IGHV3-72*01 F	92.86		NS	K
**PT29**	F	0	Stable	0	Homsap IGHV3-7*01 F	90.97		NS	L
**PT30**	F	0	Stable	NA	IGHV1-46*01 F or IGHV1-46*03 F	93.4	del(13q)	NS	NA
**PT31**	M	0	Stable	0	Homsap IGHV4-34*01 F	94.4		#4	K
**PT32**	M	0	Stable	19.1	IGHV3-30*03 F, or IGHV3-30*18 F or IGHV3-30-5*01 F	95.14		#2	K
**PT33**	M	0	Stable	0	na	92.71		NA	NA
**PT34**	M	0	Stable	0	IGHV2-5*02 F	92.44		NS	K
**PT35**	M	NA	Stable	0	IGHV3-66*01 F or IGHV3-66*04 F	94		NS	K
**PT36**	M	0	Stable	0	nA	Mutated	del(13q)	NA	NA
**PT37**	F	I	Stable	0	IGHV5-10-1*03 F	99.31		NS	L
**PT38**	M	0	Stable	75.5	NA	Mutated		NA	K
**PT39**	F	0	Stable	NA	NA	Unmutated		NA	L
**PT40**	M	NA	Progressive	11.6	IGHV1-69*01 F or IGHV1-69D*01 F	100		#3	K
**PT41**	M	NA	Stable	NA	NA	NA		NA	K
**PT42**	F	0	Stable	0	NA	NA		NA	K
**PT43**	F	0	Stable	2.5	IGHV3-23*01 F, or IGHV3-23D*01 F	93.4	tris(12) del(13q)	NS	L
**PT44**	M	1	Stable	NA	IGHV5-10*03 F	98.26		#1	K
**PT45**	M	0	Stable	NA	Homsap IGHV4-59*11 F	93.68		#77	K
**PT46**	F	0	Stable	0.1	Homsap IGHV4-34*01 F, or Homsap IGHV4-34*12 F	89.47		NS	K
**PT47**	M	0	Stable	0.1	IGHV1-3*01 F	88.54		NS	NA
**PT48**	F	0	Stable	0	NA	NA		NA	L
**PT49**	M	0	Stable	0	IGHV2-5*02 F	92.44		NS	K

NA = not available, NS = no subset

Disease Models & Mechanisms • Supplementary information

Table S2. Resources and reagents.

ANTIBODIES – DYES - ASSAYS	CAT NUMBER	USAGE (dilution)	SOURCE
Purified Mouse anti-human CD45	555-480	1:100 in blocking solution	BD Pharmingen
Mouse anti-human CD68	14-0688-82	1: 50 in blocking solution	Thermo Fisher Scientific
Anti-human CD31 antibody- PE, REAfinity™	130-110-807	2μl/sample	Miltenyi Biotec
Rabbit anti-human CD31	ab32457	1: 100 in blocking solution	Abcam
Anti-human CD3-ECD	A07748	5μl/sample	Beckman Coulter
Anti-human CD4-FITC	A07750	5μl/sample	Beckman Coulter
Anti-human CD8-PC7	737661	5μl/sample	Beckman Coulter
Anti-human CD3-FITC	A07746	5μl/sample	
Anti-human CD19-ECD	A07770	5μl/sample	Beckman Coulter
Anti-human CD5-PC7	A21690	5μl/sample	Beckman Coulter
Hyaluronic Acid Binding Protein, Bovine Nasal Cartilage, Biotinylated	385911-50UG	1:250 in blocking solution	Merck, Sigma-Aldrich
Collagen IV Monoclonal Antibody (1042), Alexa Fluor™ 488, eBioscience™	53-9871-82	1:50 in blocking solution	Thermo Fisher Scientific
Fibronectin Monoclonal Antibody (FN-3), Alexa Fluor™ 488, eBioscience™	53-9869-82	1:100 in blocking solution	Thermo Fisher Scientific
Anti podoplanin (PDPN)	ab10288	1: 50 in blocking solution	Abcam
Anti-human CD184-PE	555974	10μl/sample	BD Pharmingen
Anti human Cleaved Cas3	9661	1:400 in blocking solution	Cell Signaling Technology
Donkey anti-Rabbit IgG (H+L) Highly Cross-Adsorbed Secondary Antibody, Alexa Fluor™ Plus 488	A32790	1:500 in blocking solution	Thermo Fisher Scientific
Donkey anti-Mouse IgG (H+L) Highly Cross-Adsorbed Secondary Antibody, Alexa Fluor™ Plus 488	A32766	1:500 in blocking solution	Thermo Fisher Scientific
Goat anti-Rabbit IgG (H+L) Highly Cross-Adsorbed Secondary Antibody, Alexa Fluor™ Plus 647	A32733	1:500 in blocking solution	Thermo Fisher Scientific
Goat anti-Mouse IgG (H+L) Highly Cross-Adsorbed Secondary Antibody, Alexa Fluor™ Plus 647	A32728	1:500 in blocking solution	Thermo Fisher Scientific
Streptavidin Alexa Fluor 633	S21374	1:400 in blocking solution	Thermo Fisher Scientific
Streptavidin Alexa Fluor 568	S11226	1:400 in blocking solution	Thermo Fisher Scientific
**CHEMICALS, SOLUTIONS AND CULTURE MEDIA**	**cat number**		**SOURCE**
Alexa Fluor 568 Phalloidin	94072	1:250 in blocking solution	Thermo Fisher Scientific
Hoechst 33342	H1399	1:1000 in PBS	Thermo Fisher Scientific
Trypan Blue	302643		Merck, Sigma-Aldrich
Liberase™ TM Research Grade (25 μg/ml)	5401119001	25μg/ml	Roche, Merck
Liberase™ TL Research Grade (25 μg/ml)	5401020001	25μg/ml	Roche, Merck
Hyaluronidase from bovine testes	H4272-30MG	300μg/ml	Merck, Sigma-Aldrich
EGM™-2 Bullet kit	CC-3162		Lonza-Euroclone
AIM-V Medium (1X)	12055091		Gibco
DMEM High Glucose w/ L-Glutamine w/ Sodium Pyruvate	ECM0728L		Lonza-Euroclone
Fibroblast Medium (FM)	2301		ScienceCell-Clinisciences
Human Serum - Type AB male.OFF-THE-CLOT, HIV, HBsAg and HCV tested	ECS0219D		Lonza-Euroclone
RosetteSep B lymphocyte enrichment kit	15263		Stemcell Technologies
ODN 2006 (ODN 7909)	TLRL-2006-1	1μg/ml	InvivoGen
Human Interleukin-2 (hIL-2)	200-02	500 U/mL	Peprotech
ABT-199 10mg	CAY-16233-10	2,5ng/ml	Cayman Chemical
Ibrutinib	S2680	1μg/ml	Selleckchem
Fixable Viability Dye eFluor™ 660	65-0864-18	1μl/ml following instruction manual	eBioscience™-ThermoFisher Scientific
AnnexinV-FITC/Propidium Iodide (PI) Apoptosis Detection kit	88-8005-74	follow instruction manual	eBioscience™-ThermoFisher Scientific
Vybrant™ CFDA-SE Cell Tracer Kit	V12883	0,5μM	ThermoFisher Scientific
CellTrace Violet™ proliferation Kit	C34557	5μM	ThermoFisher Scientific
**INSTRUMENTS and other materials**	**cat number**		**SOURCE**
Cellstar 96well suspension culture plates	650185		Greiner Bio-one
Rotary Cell culture system bioreactor (RCCS™)	RCCS-4DQ		Synthecon, USA
10ml disposable vessels	D-410		Synthecon, USA
Gelatin sponge, Spongostan™	MS0005/J&J		Ethicon, Inc, USA

Disease Models & Mechanisms • Supplementary information
